# The Chloroplast Genome of *Passiflora edulis* (Passifloraceae) Assembled from Long Sequence Reads: Structural Organization and Phylogenomic Studies in Malpighiales

**DOI:** 10.3389/fpls.2017.00334

**Published:** 2017-03-10

**Authors:** Luiz A. Cauz-Santos, Carla F. Munhoz, Nathalie Rodde, Stephane Cauet, Anselmo A. Santos, Helen A. Penha, Marcelo C. Dornelas, Alessandro M. Varani, Giancarlo C. X. Oliveira, Hélène Bergès, Maria Lucia C. Vieira

**Affiliations:** ^1^Departamento de Genética, Escola Superior de Agricultura “Luiz de Queiroz”, Universidade de São Paulo, PiracicabaBrazil; ^2^Institut National de la Recherche Agronomique, French Plant Genomic Resource Center, Castanet-TolosanFrance; ^3^FuturaGene Brasil Tecnologia Ltda., São PauloBrazil; ^4^Departamento de Tecnologia, Faculdade de Ciências Agrárias e Veterinárias, Universidade Estadual Paulista, JaboticabalBrazil; ^5^Departamento de Biologia Vegetal, Instituto de Biologia, Universidade Estadual de Campinas, CampinasBrazil

**Keywords:** chloroplast genome, *Passiflora*, single molecule real-time (SMRT) sequencing, phylogenomics, Malpighiales, Fabids

## Abstract

The family Passifloraceae consists of some 700 species classified in around 16 genera. Almost all its members belong to the genus *Passiflora*. In Brazil, the yellow passion fruit (*Passiflora edulis*) is of considerable economic importance, both for juice production and consumption as fresh fruit. The availability of chloroplast genomes (cp genomes) and their sequence comparisons has led to a better understanding of the evolutionary relationships within plant taxa. In this study, we obtained the complete nucleotide sequence of the *P. edulis* chloroplast genome, the first entirely sequenced in the Passifloraceae family. We determined its structure and organization, and also performed phylogenomic studies on the order Malpighiales and the Fabids clade. The *P. edulis* chloroplast genome is characterized by the presence of two copies of an inverted repeat sequence (IRA and IRB) of 26,154 bp, each separating a small single copy region of 13,378 bp and a large single copy (LSC) region of 85,720 bp. The annotation resulted in the identification of 105 unique genes, including 30 tRNAs, 4 rRNAs, and 71 protein coding genes. Also, 36 repetitive elements and 85 SSRs (microsatellites) were identified. The structure of the complete cp genome of *P. edulis* differs from that of other species because of rearrangement events detected by means of a comparison based on 22 members of the Malpighiales. The rearrangements were three inversions of 46,151, 3,765 and 1,631 bp, located in the LSC region. Phylogenomic analysis resulted in strongly supported trees, but this could also be a consequence of the limited taxonomic sampling used. Our results have provided a better understanding of the evolutionary relationships in the Malpighiales and the Fabids, confirming the potential of complete chloroplast genome sequences in inferring evolutionary relationships and the utility of long sequence reads for generating very accurate biological information.

## Introduction

Malpighiales is an order of flowering plants that belongs to the clade Eurosids I, also known as Fabids (The Angiosperm Phylogeny Group, 2009). This large order includes 42 families, more than 700 genera, and contains approximately 16,000 species forming an extremely diverse group of plants in terms of their morphological and ecological aspects (The Angiosperm Phylogeny Group, 2009; Wurdack and Davis, 2009). The Passifloraceae family is a member of the Malpighiales (Judd et al., 2008) and consists of some 700 species of herbaceous or woody vines, shrubs and trees, classified in around 16 genera, and almost all its members belong to the large and variable genus *Passiflora*, popularly known as passion flowers or passion fruits (Feuillet, 2004). There are around 520 species of *Passiflora*, the majority of which are distributed pantropically; the most derived and specialized species are distributed in the Neotropics and Africa (Ulmer and MacDougal, 2004).

The genus *Passiflora* has long attracted considerable attention due to its economic value, broad geographic distribution, and remarkable species diversity, particularly with regard to flower morphology. The main economic value lies in the production of passion fruit juice, an essential exotic ingredient in juice blends. Furthermore, some species are of great ornamental value or used in phytotherapeutic remedies (Ramaiya et al., 2014). *Passiflora* seed oil is also well-suited for use as a regenerative ingredient in cosmetics products.

In Brazil, passion fruit cultivation began relatively recently and has earned the country an outstanding position as the world’s top producer of yellow passion fruit (*Passiflora edulis*). It is an outcrossing, diploid (*n* = 9) (Cuco et al., 2005) species with perfect self-incompatibility (Bruckner et al., 1995; Rêgo et al., 2000), and insect-pollinated flowers. Genetic (Moraes et al., 2005; Oliveira et al., 2008) and molecular-based studies have been carried out in our laboratory (Santos et al., 2014; Munhoz et al., 2015), which is able to satisfy the needs of a wide range of breeders to boost passion fruit crop production and fruit quality.

The nuclear genome size of *P. edulis* (expressed in 1C) was estimated at 1.258 (Yotoko et al., 2011) or 1,595 pg (Souza et al., 2004). More recently, a very efficient strategy for obtaining initial insight into the content of this particular genome involved the sequencing of the terminal regions (BAC-ends) of a representative number of BAC clones selected at random from a *P. edulis* genomic library (Santos et al., 2014). This library was constructed by and deposited at INRA-CNRGV and covers around six times the genome length of *P. edulis*^1^. Our group was able to characterize some 10,000 high-quality sequences (100 to 1,255 bp) and identify reads likely to contain repetitive mobile elements and SSRs, and to estimate the GC-content of the reads. Approximately one 10th of the BAC end-sequences contained protein sequences, and gene ontology terms were assigned to most of them. Finally, we were able to map a number of BAC-end pair sequences to intervals of *Arabidopsis thaliana*, *Vitis vinifera* and chiefly to *Populus trichocarpa* chromosomes, representing regions of potential microsynteny. Additionally, a number of BAC clones were identified as containing chloroplast DNA sequences (Santos et al., 2014).

The chloroplast genome usually occurs in multiple copies within the organelle. It consists of fairly long circular or linear DNA molecules, normally ranging from 120 to 180 kb in angiosperms. It has a quadripartite structure characterized by the presence of two copies of a large IRA and IRB separating a SSC and a LSC region. There is a typical gene partitioning pattern with about 80 protein coding genes in addition to tRNA and rRNA coding genes (Sugiura, 1992; Yang et al., 2010). It also contains 20 group II introns (Barkan, 2004), that derived from a class of mobile elements that are thought to be ancestors of spliceosomal introns and eukaryotic retrotransposons (Lambowitz and Zimmerly, 2011). Though highly conserved, changes in the composition of chloroplast genomes may occur, and rearrangements or even gene losses have been documented (Tangphatsornruang et al., 2011; Li et al., 2013).

Chloroplast DNA, specifically non-coding sequences, has been used extensively to investigate phylogenetic relationships in plants (Shaw et al., 2005), including *Passiflora* species (Muschner et al., 2003; Yockteng and Nadot, 2004). Chloroplast genes such as *rbcL*, *matK*, *ndhF*, *atpB*, and *rps2* have been used in evolutionary studies at higher taxonomic levels. Currently, it is possible to generate entire chloroplast genomes as well as entire chloroplast gene sequences and both can be used simultaneously to determine phylogenies (Martin et al., 2013). For instance, the relationships between wild and domestic species within the genus *Citrus* were elucidated based on a phylogenetic analysis of 34 entire chloroplast genomes (Carbonell-Caballero et al., 2015). Very recently, the complete chloroplast genome sequences were used to infer phylogenetic relationships in the *Quercus* genus (Yang et al., 2016).

The development of NGS technologies has provided highly efficient, low-cost DNA sequencing platforms that produce large volumes of short reads (Shin et al., 2013). However, more recently, third generation sequencing technologies producing longer DNA reads have begun to emerge, including the Pacific Biosciences single molecule real-time (SMRT) sequencer that became available in 2011^2^. For example, using PacBio sequence data it was possible to generate an entire chloroplast genome assembled into a single large contig with a high degree of accuracy and at a much greater depth of coverage due to longer read lengths (Ferrarini et al., 2013).

In this study, we present the complete nucleotide sequence and the organization of the chloroplast genome of *P. edulis*, the first report on the Passifloraceae family. We were able to localize genes, introns and intergenic spacers, as well as repetitive elements, and to compare the cpDNA *of P. edulis* with that of other of phylogenetically close species, searching for syntenic regions and possible sequence rearrangements. Moreover, the order Malpighiales was investigated using the available entire chloroplast genomes of members of the four families that compose this order. Finally, a phylogenomic analysis was performed based on a set of chloroplast genes, with the aim of describing species relationships within the Fabids.

## Materials and Methods

### Plant Material

The ‘IAPAR-123’ passion fruit (*P. edulis*) accession described in Carneiro et al. (2002) was used in the present study. The other complete cp genomes and cp gene sequences were downloaded from GenBank. A list of species and GenBank accession numbers are provided in the Supplementary Table S1.

### Sequencing and Subsequent Assembly of *Passiflora edulis* Chloroplast DNA Using the Pacbio RS II Platform

The large-insert BAC library of *P. edulis* was constructed and maintained at the French Plant Genomic Resources Centre^3^. It was previously accessed using the BES approach and comparative genome mapping, and two clones (Pe69Q4G9 and Pe85Q4F4) were found to match the *Arabidopsis thaliana* chloroplast genome (Santos et al., 2014). In the present study, these clones were selected so that their entire inserts could be sequenced. The DNA was then isolated using the Nucleobond Xtra Midi Plus kit (Macherey-Nagel, Düren, Germany) following the manufacturer’s instructions, after growing the bacterial clones on LB medium (100 mL) containing chloramphenicol as the selective marker (12.5 μg/mL).

Around 1.5 μg of each individual cpDNA were pooled together with other *P. edulis* BAC inserts (12 in total) for the construction of an SMRT library using the standard Pacific Biosciences (San Francisco, CA, USA) preparation protocol for 10 kb libraries. The pool was then sequenced in one SMRT Cell using the P4 polymerase in combination with C2 chemistry, following the manufacturer’s standard operating procedures and using the Pacific Biosciences PacBio RS II platform. Sequencing was performed by GATC Biotech^4^.

The reads were assembled following a hierarchical genome assembly process HGAP workflow (Chin et al., 2013), and using the SMRT^®^ Analysis (v2.2.0) software suite for HGAP implementation. Reads were first aligned by the PacBio long read aligner or BLASR (Chaisson and Tesler, 2012) against the complete genome of *Escherichia coli* strain K12 substrain DH10B (GenBank: CP000948.1). The *E. coli* reads, as well as low quality reads (minimum read length of 500 bp and minimum read quality of 0.80), were removed from the data set. Filtered reads were then preassembled to yield long, highly accurate sequences. To perform this step, smallest and longest reads were separated from each other to correct read errors by mapping single-pass reads to longest reads (seed reads), which represent the longest portion of the read length distribution. Next, the sequences were filtered against vector (BAC) sequences, and the Celera assembler was used to assemble data and obtain draft assemblies. The last step of the HGAP workflow is performed in order to significantly reduce the remaining InDel and base substitution errors in the draft assembly. The Quiver algorithm was used for this purpose. It is a quality-aware consensus algorithm that uses rich quality scores (QV scores) embedded in Pacific Biosciences’ bas.h5 files. Once the polished assembly was obtained, each BAC sequence was individualized by matching its paired-end sequences to the assembled sequences using BLAST. Read coverage was assessed by aligning the raw reads on the assembled sequences with BLASR.

### Obtaining the Complete Chloroplast Genome

The sequences obtained from both inserts (Pe69Q4G9 and Pe85Q4F4) were aligned using ClustalX software (Larkin et al., 2007) to obtain a single contig. Specific primers were designed at the sequence ends of this contig in order to find out whether the circular chloroplast genome was complete. PCR reactions were then performed using a 9700 thermal cycler (Applied Biosystems, Foster City, CA, USA) in reaction mixtures containing 20 ng template DNA (*P. edulis* accession ‘IAPAR-123’), 1× buffer, 1 mM MgCl_2_, 0.2 mM of each dNTP, 0.3 μM of the forward and reverse primers, 1.2 U Go Taq Flex DNA polymerase (Promega, Madison, WI, USA), and ultra-pure water to bring the final volume up to 20 μL. The thermal profile for amplification was: 95°C for 5 min, 35 cycles at 95°C for 40 s, 55°C for 40 s and 72°C for 1 min, followed by a final 8 min incubation at 72°C. The amplified fragments were checked on 1% (w/v) agarose gel with a 100 bp molecular size standard Invitrogen (Carlsbad, CA, USA). The PCR product was purified using the Wizard^®^ SV Gel kit and PCR Clean-Up System (Promega), and then used as a template for the sequencing reaction based on the Sanger method. It was subjected to capillary electrophoresis in the ABI Prism 3100 sequencer (Applied Biosystems). The sequence of the PCR product was aligned with the single contig to obtain the complete sequence of the cp genome.

#### Genome Annotation

The cp genome was preliminarily annotated using the DOGMA (Dual Organellar GenoMe Annotator) online program (Wyman et al., 2004), with default settings to identify coding sequences (CDS), rRNAs and tRNAs based on the Plant Plastid Code and BLAST homology searches, followed by manual corrections for start and stop codons, and intron positions. All tRNA genes were further confirmed using the tRNAscan-SE online search server. Pseudogenes were classified based on the loss of parts in their sequences or by the presence of internal stop codons. The circular genome map was designed by the GenomeVx program (Conant and Wolfe, 2008). Codon usage frequencies and the relative synonymous codon usage (RSCU) were calculated for all exons of the protein-coding genes using DAMBE 5 (Xia, 2013). Pseudogenes were not included in this analysis.

#### Comparative Analysis of Chloroplast Genomes

To examine the expansion of the IR borders, the IR-LSC and IR-SSC boundaries with full annotations for the adjacent genes were manually analyzed across 11 sequenced species related to *P. edulis*, totalling 12 comparisons. These species are members of the families that compose the order Malpighiales: *P. edulis* (Passifloraceae), *P. trichocarpa* and *Salix purpurea* (Salicaceae), *Hevea brasiliensis*, *Manihot esculenta*, *Jatropha curcas* and *Ricinus communis* (Euphorbiaceae), *Hirtella physophora*, *Licania heteromorpha*, *Couepia guianensis*, *Parinari campestris* and *Chrysobalanus icaco* (Chrysobalanaceae) (Supplementary Table S1).

In addition, a multiple alignment with all available entirely sequenced cp genomes of Malpighiales species (22 in the total) (Supplementary Table S1), including *P. edulis*, was run in progressive Mauve v.2.4.0 (Darling, 2004). Briefly, this method identifies conserved genomic regions, rearrangements and inversions in conserved regions, and the sequence breakpoints of these rearrangements across multiple genomes.

Next, to validate the three inversions found in the passion fruit cp genome, a pair of primers was designed to anneal the 5′- and 3′ ends of the boundaries of each inversion. The amplicons of the expected size and the corresponding sequences should cover each border both downstream and upstream. PCR and Sanger sequencing were performed as described above.

#### Identification of Repeated Elements

REPuter (Kurtz et al., 2001) was used to identify direct and palindromic repeated elements, based on the following criteria: minimum repeat size ≥ 30 bp and sequence identities ≥ 90% (Hamming distance equal to 3).

Microsatellites or SSRs that consist of tandemly arranged repeats of short DNA motifs (1–6 bp in length) were predicted using MISA (MIcroSAtellite)^5^. This tool allows the identification and localization of microsatellites in genome sequences, including organelle genome sequences. The criteria used to search SSR motifs were set as follows: motifs between one and six nucleotides long, with a minimum repeat number defined as 10, 5, and 4 units for mono-, di-, and trinucleotide SSRs, respectively, and three units for each tetra-, penta-, and hexanucleotide SSRs.

### Phylogenomic Studies

We performed two phylogenomic studies. The first was restricted to the Malpighiales based on the available entire chloroplast genomes of members of the four families that compose this order (22 species in total, Supplementary Table S1). In the second, a set of chloroplast genes was used to infer the relationships within the Fabids (42 species in total, Supplementary Table S1).

Entire cloroplastidial genomes of Malpighiales representing the families Passifloraceae, Salicaceae, Euphorbiaceae, and Chrysobalanaceae were used as the ingroup in phylogenomic comparisons, and the cp genome of *V. vinifera* (Vitaceae, Vitales) was used as the outgroup in order to root the phylogenetic tree. The data set consisting of 23 taxa was aligned at nucleotide level in server-based program MAFFT version 7.221, using the FFT-NS-2 algorithm with default settings. To generate the alignment, inverted sequences detected in the cp genomes of *P. edulis* and *H. brasiliensis* were reversed and the respective positions adjusted. The raw alignment was manually corrected in BioEdit (Hall, 1999) and further processed in GBLOCKS 0.91b (Castresana, 2000) in order to remove regions containing gap positions, with a minimum block length of five, and maximum number of contiguous non-conserved positions of eight. The resulting alignment was analyzed in jModelTest software version 2.1.7 (Darriba et al., 2012) to determine the optimal model of molecular evolution and gamma rate heterogeneity using the AIC.

Maximum likelihood analysis was performed using PAUP version 4.0a146 (Swofford, 2002), based on the transversional (TVM) substitution model, gamma distribution of rate heterogeneity with five discrete categories (+G). To estimate the level of support for the ML topology, bootstrap analysis was performed on 1,000 replicates. BI was run in MrBayes, version 3.1.2 (Ronquist and Huelsenbeck, 2003) but based on the GTR +G model, the second model chosen by jModelTest taking the AIC values into account. The Markov chain Monte Carlo (MCMC) algorithm was run for 5,000,000 generations, sampling one tree every 100 generations. The first 25% of trees were discarded as burn-in to estimate the values of posterior probabilities. Convergence diagnostics were monitored on the basis of an average standard deviation of split frequencies below 0.01, potential scale reduction factor (PSRF) values close to 1.0, and ESS values above 200. Trees were visualized using FigTree version 1.4.0.

A set of 43 nucleotide sequences of homologous protein-coding chloroplast genes from 42 species representing all orders of the Fabids clade (Rosales, Fagales, Cucurbitales, Fabales, Malpighiales, Celastrales, Oxalidales, Zygophillales) were used as the ingroup in the phylogenomic comparisons. *V. vinifera* (Vitaceae, Vitales) was chosen to serve as outgroup to produce a rooted tree. A list of the chloroplast gene sequence sources is provided in Supplementary Table S1.

First, each protein-coding gene sequence was aligned using ClustalW with default settings and the raw alignments manually corrected in program BioEdit (Hall, 1999) and further processed in GBLOCKS (Castresana, 2000), excluding gap positions from the data set, with a minimum block length of five, and a maximum number of contiguous non-conserved positions of eight. Next, all individual filtered alignments were concatenated into a single alignment matrix. Both conserved and variable nucleotide positions in the alignment matrix were analyzed in MEGA6 (Tamura et al., 2013).

jModelTest software version 2.1.7 (Darriba et al., 2012) was used to determine the optimal model of molecular evolution and gamma rate heterogeneity based on AIC. Both ML and BI were used to infer the phylogenomic relationships within the Fabids clade. ML analysis was performed using RAxML version 8.2.4 (Stamatakis, 2014). The GTR model of nucleotide substitution was selected for ML analysis, taking into account the gamma distribution of rate heterogeneity with five discrete categories (+G). To estimate the support of the ML topology, a bootstrap analysis was performed on 1,000 replicates. BI was run on MrBayes software, version 3.1.2 (Ronquist and Huelsenbeck, 2003) with the GTR +G model. The MCMC algorithm ran for 5,000,000 generations, sampling one tree every 100 generations. The first 25% of trees were discarded as burn-in to estimate the values of posterior probabilities. Convergence diagnostics were monitored on the basis an average standard deviation of split frequencies below 0.01, PSRF values close to 1.0, and ESS values above 200. Trees were visualized using FigTree version 1.4.0.

## Results And Discussion

### Data Output from the PacBio RS II Platform and Assembly of Chloroplast Genome Sequences

Following extraction of reads containing only chloroplast genome sequence data and subsequent error correction, 2,340 PacBio RS reads from the clone insert Pe69Q4G9 were recovered, ranging from 500 to 22,458 bp, and containing a total of 94,052 bp assembled into a contig. The average depth of coverage of the consensus sequence was 96× and the average GC content was 37%. The final quality of the assembly corresponded to a nominal QV of 48.48 (approximately 99.999% accuracy). Similarly, 4,972 reads from the clone insert Pe85Q4F4 were recovered, ranging from 500 to 26,035 bp, and containing a total of 91,155 bp assembled into a contig. The average depth of coverage of the consensus sequence was 172× and the average GC content was 38%. The final quality of the assembly corresponded to a QV of 48.57.

It is worth noting the usefulness of the HGAP workflow as a solution for successfully resolving long repeat regions, as already pointed out by Chin et al. (2013). The high quality sequence data generated by the PacBio RS II Platform, added to its capability to assemble long reads, allowed us to obtain a single contig for each clone insert and then the complete cp genome of *P. edulis*. Both contigs were aligned and merged into a single long contig of 151,362 bp with an overlapping region of 33,848 bp. The amplification reaction using the primer pair designed to anneal at the extremities of the large contig and genomic DNA from *P. edulis* accession ‘IAPAR-123’ as a template produced an amplicon of 325 bp, which was subsequently sequenced and aligned with the long contig sequence. Thus, a 44-nucleotide sequence was obtained and added for closing the gap to produce the complete sequence of the circular molecule of 151,406 bp. The chloroplast genome sequence of *P. edulis* has been submitted to the NCBI GenBank and has received the accession number KX290855.

The chloroplast genomes of *Potentilla micrantha* (Ferrarini et al., 2013) and *Aconitum barbatum* var. *puberulum* (Chen et al., 2015) were entirely sequenced using the PacBio RS II Platform, and both studies described the advantages of using this method. For instance, Ferrarini et al. (2013) highlight the generation of a single contig covering the entire cp genome of *P. micrantha*, whereas Illumina HiSeq2000 sequencing data have lower genome coverage (some regions have zero or very low coverage) and the resulting assembly consisted of seven contigs. Similarly, Chen et al. (2015) emphasize the low level of errors (~0.0027%) in PacBio reads, since after applying the necessary filter, the result is an error-corrected consensus read with a higher intra-molecular accuracy. Interestingly, even in the absence of any other biological information on the target species, the authors stated that it took less than half an hour to finish the genome assembly step, confirming that long read lengths are superior, especially for *de novo* assemblies.

### Organization of the *P. edulis* Chloroplast Genome

**FIGURE 1:**
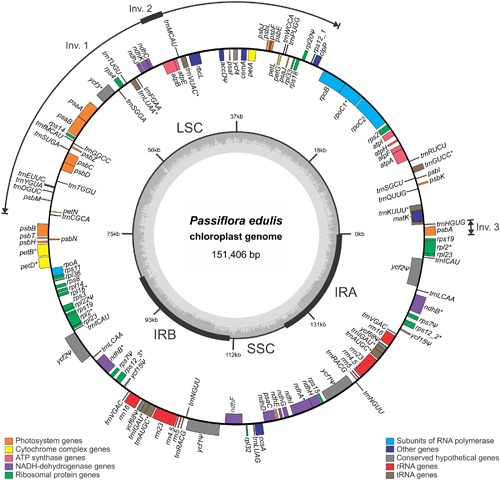
***Passiflora edulis* chloroplast genome map.** Genes are represented as boxes inside or outside the large circle to indicate clockwise (inside) or counter clockwise (outside) transcription. The color of the gene boxes indicates the functional group to which the gene belongs. The thickened lines of the smaller circle indicate IR regions. The inner most circle denotes the GC content across the genome. LSC, large single-copy region; SSC, small single-copy region. Intron-containing genes are marked with ‘*’; Pseudogenes are marked with ‘Ψ.’ Inv., inversion.

Chloroplast genome annotation resulted in the identification of 105 unique genes, including 30 tRNAs, 4 rRNAs and 71 protein coding genes (**Table 1**). The molecule has a typical quadripartite structure characterized by the presence of two copies of an IRA and IRB each of 26,154 bp (34.6% in total) separating a SSC region of 13,378 bp (8.8%) and a LSC region of 85,720 bp (56.6%). Genes arising from duplication events created perfect IRs, each containing the same 16 genes, totalling 120 genes in the complete chloroplast DNA molecule (**Figure 1**).

**Table 1:** Gene content of the *Passiflora edulis* chloroplast genome according to respective categories.

Category	Gene
Subunits of photosystem I	*psaA*, *psaB*, *psaC*, *psaI*, *psaJ*
Subunits of photosystem II	*psbA*, *psbB*, *psbC*, *psbD*, *psbE*, *psbF*, *psbH*, *psbI*, *psbJ*, *psbK*, *psbL*, *psbM*, *psbN*, *psbT*, *psbZ*
Subunits of cytochrome b/f complex	*petA*, *petB*^a^, *petD*^a^, *petG*, *petL*, *petN*
Subunits of ATP synthase	*atpA*, *atpB*, *atpE*, *atpF*, *atpH*, *atpI*
Large subunit of rubisco	*rbcL*
Subunits of NADH-dehydrogenase	*ndhA*^a^, *ndhB*^a,b^, *ndhC*, *ndhD*, *ndhE*, *ndhF*, *ndhG*, *ndhH*, *ndhI*, *ndhJ*, *ndhK*
Proteins of large ribosomal subunit	*rpl2*^a,b^, *rpl14*, *rpl16*^a^, *rpl23*^b^, *rpl32*, *rpl33*, *rpl36*
Proteins of small ribosomal subunit	*rps2*, *rps3*, *rps4*, *rps8*, *rps11*, *rps12*^a,b,c^, *rps14*, rps15, *rps18*, *rps19*^b^
Subunits of RNA polymerase	*rpoA*, *rpoB*, *rpoC1*^a^, *rpoC2*
Cytochrome c biogenesis	*ccsA*
Maturase	*Matk*
Protease	*clpP*
Envelope membrane protein	*cemA*
Conserved hypothetical genes	*ycf3*^a^, *ycf4*
Ribosomal RNAs	*rrn4*.5^b^, *rrn5*^b^, *rrn16*^b^, *rrn23*^b^
Transfer RNAs	*trnA*-*UGC*^a,b^, *trnC*-*GCA*, *trnD*-*GUC*, *trnE*-*UUC*, *trnF*-*GAA*, *trnfM*-*CAU*, *trnG*-*UCC*^a^, *trnG*-*GCC*, *trnH*-*GUG*, *trnI*-*CAU*^b^, *trnI*-*GAU*^a,b^, *trnK*-*UUU*^a^, *trnL*-*CAA*^b^, *trnL*-*UAA*^a^, *trnL*-*UAG*, *trnM*-*CAU*, *trnN*-*GUU*^b^, *trnP*-*UGG*, *trnQ*-*UUG*, *trnR*-*ACG*^b^, *trnR*-*UCU*, *trnS*-*GCU*, *trnS*-*GGA*, *trnS*-*UGA*, *trnT*-*GGU*, *trnT*-*UGU*, *trnV*-*GAC*^b^, *trnV*-*UAC*^a^, *trnW*-*CCA*, *trnY*-*GUA*

*^a^Intron-containing gene; ^b^two gene copies in the IRs; ^c^gene divided into two independent transcription units.*

Protein coding genes constitute 37.3% of the chloroplast genome (75 genes, totalling 56,428 bp), in addition to tRNA and rRNA coding genes that constitute 1.8% (37 genes totalling 2,801 bp) and 6.0% (eight genes totalling 9,048 bp) respectively of the chloroplast genome. Introns constitute 10.7% (totalling 16,155 bp), while intergenic regions and pseudogenes form the remaining 44.2% (totalling 66,974 bp). The amount of GC was estimated at 37%.

tRNA genes are spread throughout the genome and encode the 20 amino acids incorporated into proteins. One is present in the SSC region, 21 in the LSC, and 7 in the IRs, which also have 5 protein-coding genes and 4 rRNA genes. A total of 18,799 codons represent the coding capacity of the 71 protein-coding genes (**Table 2**). The most frequent amino acid was found to be leucine (2,026 codons corresponding to 10.8% of total DNA) and the least frequent cysteine (218 codons corresponding to 1.2% of total DNA).

**Table 2:** Codon usage and codon–anticodon recognition pattern of the *Passiflora edulis* chloroplast genome.

Codon	Amino acid	Number	RSCU^a^	%^b^	tRNA	Codon	Amino acid	Number	RSCU^a^	%^b^	tRNA
GCU	A	513	1.727	43.2		CCA	P	225	1.108	27.7	*trnP-UGG*
GCG	A	150	0.505	12.6		CCC	P	158	0.778	19.5	
GCC	A	206	0.694	17.3		CCU	P	325	1.601	40.0	
GCA	A	319	1.074	26.9	*trnA-UGC*^c^	CCG	P	104	0.512	12.8	
UGU	C	162	1.486	74.3		CAA	Q	523	1.59	79.5	*trnQ-UUG*
UGC	C	56	0.514	25.7	*trnC-GCA*	CAG	Q	135	0.41	20.5	
GAU	D	524	1.53	76.5		AGA	R	302	1.529	28.5	*trnR-UCU*
GAC	D	161	0.47	23.5	*trnD-GUC*	AGG	R	93	0.471	8.8	
GAG	E	202	0.441	22.0		CGA	R	250	1.508	23.6	
GAA	E	715	1.559	78.0	*trnE-UUC*	CGC	R	80	0.483	7.6	
UUU	F	716	1.351	67.5		CGG	R	88	0.531	8.3	
UUC	F	344	0.649	32.5	*trnF-GAA*	CGU	R	245	1.478	23.2	*trnR-ACG*
GGU	G	471	1.323	33.1		AGC	S	107	0.542	7.8	*trnS-GCU*
GGG	G	233	0.654	16.4		AGU	S	288	1.458	21.1	
GGC	G	169	0.475	11.9		UCA	S	256	1.053	18.7	*trnS-UGA*
GGA	G	551	1.548	38.7	*trnG-GCC*	UCC	S	223	0.918	16.3	*trnS-GGA*
CAC	H	102	0.462	23.1	*trnH-GUG*	UCG	S	114	0.469	8.3	
CAU	H	340	1.538	76.9		UCU	S	379	1.56	27.7	
AUU	I	834	1.515	50.5		ACC	T	170	0.688	17.2	*trnT-GGU*
AUA	I	506	0.919	30.6	*trnI-CAU*	ACA	T	308	1.247	31.2	*trnT-UGU*
AUC	I	311	0.565	18.8	*trnI-GAU*^c^	ACG	T	97	0.393	9.8	
AAA	K	704	1.529	76.4	*trnK-UUU*^c^	ACU	T	413	1.672	41.8	
AAG	K	217	0.471	23.6		GUU	V	388	1.399	35.0	
CUA	L	278	1.214	13.7	*trnL-UAG*	GUG	V	145	0.523	13.1	
CUC	L	120	0.524	5.9		GUC	V	139	0.501	12.5	*trnV-GAC*
CUG	L	97	0.424	4.8		GUA	V	437	1.576	39.4	*trnV-UAC*^c^
CUU	L	421	1.838	20.8		UGG	W	327	1	100	*trnW-CCA*
UUA	L	735	1.324	36.3	*trnL-UAA*^c^	UAC	Y	124	0.363	18.2	*trnY-GUA*
UUG	L	375	0.676	18.5	*trnL-CAA*	UAU	Y	559	1.637	81.8	
AUG	M	464	1	100	*trn(f)M-CAU*	UGA	*	15	0.584	19.5	
AAC	N	186	0.464	23.2	*trnN-GUU*	UAG	*	24	0.935	31.2	
AAU	N	615	1.536	76.8		UAA	*	38	1.481	49.4	

^a^Relative synonymous codon usage; ^b^codon frequency (in %) per amino acid; ^c^intron-containing tRNA genes. *Stop codon.

Fifteen unique genes (nine protein- and six tRNA-coding genes) have introns, and two introns were found in only one gene, *ycf3*. The largest intron occurs in the *trnK-UUU* gene (2,524 bp) in which the *matK* gene (1,506 bp) is inserted, and the smallest occurs in the *rps12* gene (537 bp). The number of introns identified in the cp genome of *P. edulis* is similar to that of other species. For instance, in *S. purpurea* (Wu, 2015) there are 17 intron-containing genes, three of them containing two introns, including *ycf3.* Similar figures have been found for *R. communis* (Rivarola et al., 2011), whereas in *H. brasiliensis* (Tangphatsornruang et al., 2011), 23 introns are inserted in 22 cp genes.

The *matK* gene was found in the intronic region of the *trnK-UUU* gene in *P. trichocarpa* (Tuskan et al., 2006), *H. brasiliensis* (Tangphatsornruang et al., 2011), and *M. esculenta* (Daniell et al., 2008). This gene encodes the unique maturase found in the plastid genomes of land plant species, but it does not contain the reverse transcriptase domain and therefore is not able to promote intron mobility. The *clpP* gene has no intron in *P. edulis*, although it does have two introns in the following conordinal-related species: *P. trichocarpa* (Tuskan et al., 2006), *S. purpurea* (Wu, 2015), *H. brasiliensis* (Tangphatsornruang et al., 2011), *M. esculenta* (Daniell et al., 2008), *J. curcas* (Asif et al., 2010), and *R. communis* (Rivarola et al., 2011). Similar intron losses have already been reported in legumes, almost exclusively in the clade known as the IR-lacking clade (IRLC) (Jansen et al., 2008). Coincidently, the *clpP* gene is located at the beginning of first the inversion found in the *P. edulis* cp genome (**Figure 1**), which may lead to intron losses.

Group I and II introns have been derived from mobile genetic elements, which explains why they are lost or gained in the evolution of chloroplast genomes (Barkan, 2004). Both cyanobacteria and algae, as well as land plant species, share a single group I intron in the *trnL*-UAA gene, which is therefore considered the more ancestral (Simon et al., 2003). Usually, the *atpF* gene shows a conserved group II intron; however, it is absent in the *atpF* gene of *P. edulis*. This loss was also reported in other species of *Passiflora* (Jansen et al., 2007). Daniell et al. (2008) have suggested an association between C-to-T substitutions (at nt position 92) and the loss of the intron in *M. esculenta* and in other *atpF* gene sequences of Malphigiales, implying that recombination between an edited mRNA and the *atpF* gene may be a possible mechanism for this intron loss.

Two protein-coding genes show alternative initiation codons. The ‘GTG’ triplet was found in the *rps19* and *ndhD* genes. This triplet was also found in the *rps19* gene of *J. curcas* (Asif et al., 2010), *H. brasiliensis* (Tangphatsornruang et al., 2011), and *Cynara cardunculus* (Curci et al., 2015). The *rps12* gene is a *trans*-spliced gene consisting of three exons: the first exon (5′-*rps12*) is located in the LSC region, far from the other two located in the IRs. This kind of organization was observed in *P*. *trichocarpa*, *S*. *purpurea*, *H*. *brasiliensis*, *M*. *esculenta*, *J*. *curcas*, and *R*. *communis*. Interestingly the *infA* gene, which codes for translation initiation factor 1, and the *rps16* gene, which codes for a S16 ribosomal protein are absent in the *P. edulis* cp genome, as previously reported in other species of *Passiflora* (Jansen et al., 2007). These genes were also lost or are non-functional in related species *M. esculenta* (Daniell et al., 2008), *J. curcas* (Asif et al., 2010) and in seven species of Salicaceae (Wu, 2015). It is worth noting that Passifloraceae are known to have chloroplast gene and intron losses (Hansen et al., 2006; Jansen et al., 2007; Daniell et al., 2008).

Eight pseudogenes were identified, five in the IRs and three in the LSC region. The nucleotide sequences of the *rpl22*, *ycf1*, and *ycf2* pseudogenes are smaller in length compared to those of the functional genes identified in other genomes. We found a truncated portion of the *rpl22* pseudogene that has also been identified in *Passiflora biflora*, *P. quadrangularis*, and *P. cirrhiflora* (Jansen et al., 2011), but no studies have been performed to demonstrate whether this partial copy is functional or whether there is an *rpl22* functional copy in the nucleus. There is evidence that this gene was exported to the nucleus in some Rosids (*Castanea*, *Prunus*, and *Theobroma*) and *Fagaceae*, and it is assumed that the transfer resulted from two independent events. An additional event may have occurred in *Passiflora* (Jansen et al., 2011).

Only one of the two copies of *ycf1* is a pseudogene in the *H. brasiliensis*, *M. esculenta*, *J. curcas*, and *C. cardunculus* cp genomes, as most of their sequences have been lost. Interestingly, in *P. edulis* both copies lost parts of their sequences, representing non-functional *ycf1* genes (**Figure 1**). On the other hand, the *rpl20*, *rps7*, *ycf15*, and *ycf68* pseudogenes have 5, 1, 3, and 3 internal stop codons, while the *accD* gene was found to have repetitive elements at the 5′ end.

#### Comparative Analysis of Chloroplast Genomes

**FIGURE 2:**
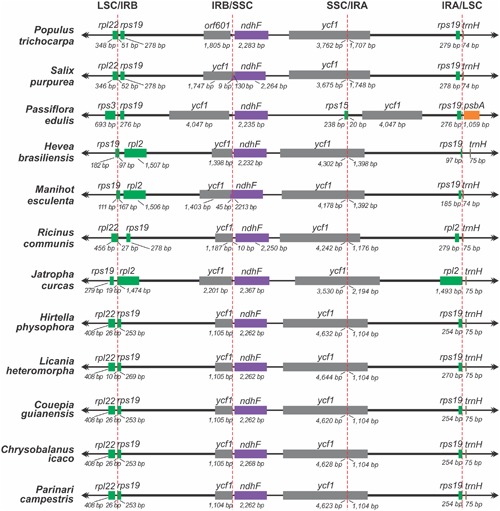
**Comparison of the border positions of LSC, SSC and IR regions among chloroplast genome sequences from 12 species of the order Malpighiales**.

As commonly done in studies of border expansion in the IRs region, the IR-LSC and IR-SSC boundaries with full annotations for the adjacent genes were reexamined across 11 sequenced species closely related to *P. edulis*. Note that the *rps15* gene of *P. edulis* is located at the end of the SSC region, expanding 20 bp toward the IRA region. It is substituted in other species by the *ycf1* gene, i.e., this gene spans SSC and IRA and there is a pseudogene copy in the IRB region. However, in *P. edulis*, both copies of the *ycf1* gene are of the same size and are full in IR regions (**Figure 2**). In addition, the *psbA* gene is the first in the LSC region due to a small inversion that is further discussed below.

**FIGURE 3:**
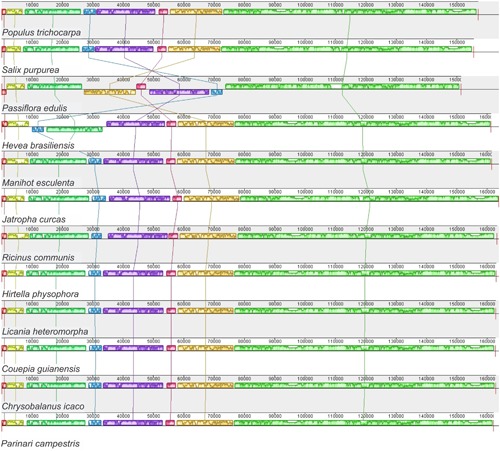
**Synteny and rearrangements detected in Malpighiales chloroplast genome sequences using the Mauve multiple-genome alignment program.** A sample of 12 species is shown. Color bars indicate syntenic blocks and connecting lines indicate correspondence blocks.

In accordance with the genome comparison results and based on the 22 members of Malpighiales with available chloroplast genomes, *P. edulis* shows a large inversion of 46,151 bp in the LSC region, between the genes *ClpP* and *trnC*-*GCA*, and a second smaller inversion of 3,765 bp between the genes *trnM*-*CAU* and *atpB*, located in the medial region of the first inversion. A third inversion of 1,631 bp is located at the beginning of the LSC region containing the *psbA* and *trnH*-*GUG* genes (**Figures 1**, **3**). All these genomic features are shown in the sequence alignment results for 12 species representing each genus of the Passifloraceae, Salicaceae, Euphorbiaceae, and Chrysobalanaceae families (**Figure 3**).

It is important to point out that the occurrence of these inversions in the cpDNA of *P. edulis* was confirmed by respective amplification reactions and subsequent Sanger sequencing of both boundaries (downstream and upstream), generating the expected sized amplicons and the corresponding sequences (Supplementary Figure S1). These sequence inversions could be typical of the *Passiflora* genus, but at present, this is pure speculation. In accordance with the alignment results (**Figure 3**), all have the same order and orientation of syntenic blocks, except for *P. edulis* and *H. brasiliensis*, in which there is an inversion of 30,000 bp in the LSC region, between the *trnE-UUC* and *trnR-UCU* genes (Tangphatsornruang et al., 2011). In conclusion, the complete chloroplast genome of *P. edulis* differs from the others because of three rearrangement events that resulted in inversions of gene block order (**Figure 3**). Otherwise, chloroplast genomes tend to be conserved and perfectly collinear, especially in the same plant family, as occurs in Salicaceae (Wu, 2015) and Chrysobalanaceae (Malé et al., 2014), also members of the Malpighiales order. The existence of rearrangements in segments of cp genomes may be useful as phylogenetic markers within genera or even within families, becoming a potential tool for understanding the evolution of plant species. Therefore, the complete sequencing of new chloroplast genomes will allow a higher accuracy in evolutionary studies of the inversions in the genus *Passiflora*.

#### Analysis of Repeated Elements

We were able to identify 36 repetitive elements, all in the LSC region. These repeats were found predominantly in intergenic regions. However, each of the members of a particular repeat was identified in the coding sequences of the *psaA* and *psaB* genes respectively; one member of other repeat is located at the beginning of the *psbI* gene sequence and the other in an intergenic region. No introns were found to contain repeated elements, and approximately 60% of repetitive elements are within a 2,513-bp region between the *accD* pseudogene and the *rbcL* gene, indicating that these elements are distributed in a peculiar arrangement in *P. edulis*. The abundance of repeated elements in this region might possibly have changed the nucleotide sequence of the *accD* pseudogene, rendering it non-functional. The repeat unit length ranged from 34 to 178 bp, and each repeat showed two copies. Forward (or direct) repeats and forward-tandem repeats (when the repeats are presented immediately one beside the other, there occurring sometimes an overlapping of both repeat units) were predominant, and only three were found to be palindromes (or reverse-complemented) (**Table 3**). Repetitive sequences are substrates for recombination and cp genome rearrangements (Milligan et al., 1989), and the number and distribution of these sequences vary from one species to another. For instance, the cp genome of *J. curcas* contains 72 repetitive sequences that are distributed in intergenic regions, introns and coding sequences (Asif et al., 2010), whereas in *V. vinifera*, 36 repetitive sequences were found, one in the coding regions of the *psaA* and *psaB* genes (Jansen et al., 2006), as in *P. edulis*, but 58% were palindromes and 12 exist in the *ycf2* gene.

**Table 3:** Type, location, and size (in bp) of repeated elements found in the *Passiflora edulis* chloroplast genome (IGS, intergenic spacer; Ψ, pseudogene).

Type	Location	Size (in bp)
Forward-tandem	IGS: *matK*-*psbK*	43
Forward	IGS: *psbK*-*psbI*, *psbI*	49
Palindrome	IGS: *psbI*-*atpA*	47
Forward-tandem	IGS: *psbI*-*atpA*	44
Palindrome	IGS: *psbI*-*atpA*	34
Forward	IGS: *rpl33*-*psaJ*, *psaI*-*accD*	47
Forward	IGS: *accD* Ψ-*rbcL*	66
Forward	IGS: *accD* Ψ-*rbcL*	58
Forward	IGS: *accD* Ψ-*rbcL*	54
Forward-tandem	IGS: *accD* Ψ-*rbcL*	41
Forward-tandem	IGS: *accD* Ψ-*rbcL*	73
Forward	IGS: *accD* Ψ-*rbcL*	61
Forward-tandem	IGS: *accD* Ψ-*rbcL*	74
Forward	IGS: *accD* Ψ-*rbcL*	39
Forward	IGS: *accD* Ψ-*rbcL*	46
Forward	IGS: *accD* Ψ-*rbcL*	56
Forward	IGS: *accD* Ψ-*rbcL*	49
Forward	IGS: *accD* Ψ-*rbcL*	67
Forward	IGS: *accD* Ψ-*rbcL*	46
Forward	IGS: *accD* Ψ-*rbcL*	65
Forward	IGS: *accD* Ψ-*rbcL*	46
Forward	IGS: *accD* Ψ-*rbcL*	43
Forward-tandem	IGS: *accD* Ψ-*rbcL*	58
Forward-tandem	IGS: *accD* Ψ-*rbcL*	124
Forward-tandem	IGS: *accD* Ψ-*rbcL*	99
Forward-tandem	IGS: *accD* Ψ-*rbcL*	74
Forward	IGS: *accD* Ψ-*rbcL*	49
Palindrome	IGS: *rbcL*-*atpE*	62
Forward-tandem	IGS: *ycf3*-*psaA*	41
Forward-tandem	IGS: *ycf3*-*psaA*	57
Forward	*psaA*, *psaB*	79
Forward-tandem	IGS: *psaB*-*rps14*	44
Forward-tandem	IGS: *petN*-*psbB*	71
Forward-tandem	IGS: *petN*-*psbB*	178
Forward	IGS: *petN*-*psbB*	117
Forward	IGS: *petN*-*psbB*	56

**FIGURE 4:**
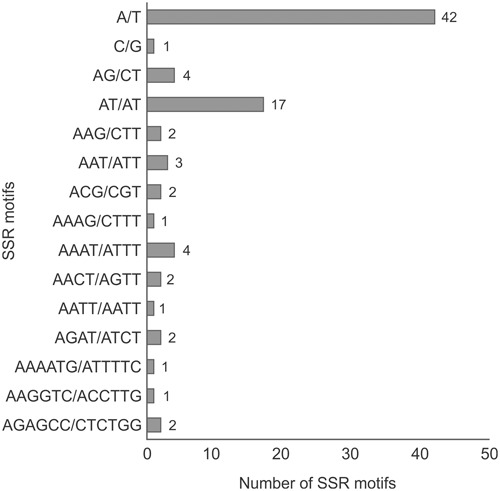
**The number of SSR motifs found in the *P. edulis* chloroplast genome, taking into account sequence complementarities.** The criteria used to search SSR motifs were set as follows: motifs between one and six nucleotides long, with a minimum repeat number defined as 10, 5, and 4 units for mono-, di-, and trinucleotide SSRs, respectively, and three units for each tetra-, penta-, and hexanucleotide SSRs.

We found 85 SSRs (microsatellites), ranging in size from 10 to 106 bp, 67 of which (79%) consisted exclusively of A/T. In terms of SSR motifs, we found 43 (50.6%) mono-, 21 (24.7%) di-, 7 (8.2%) tri-, 10 (11.8%) tetra-, and 4 (4.7%) hexanucleotides (**Figure 4**). SSRs were found mainly in non-coding regions, 68 in intergenic regions and five in pseudogenes/intronic regions. Of the 12 SSRs found in gene sequences, two interrupted elements composed of trinucleotides (GAC/ACG) were found in the *clpP* gene. In addition, the *ndhA* gene contained one mono-(A) and one tetranucleotide (AAAT), the latter also seen in the *ndhD* gene sequence. Genes *ndhF*, *rpoB*, and *rpoC1* contained one mononucleotide each (T or A), whereas *rpoC2* contained four SSRs, three mono- (T), and one dinucleotide (AT).

Simple sequence repeats were widely distributed throughout cpDNA molecules. In *P. edulis*, 55 SSRs were found in LSC, 6 in SSC, 10 in the in the IRA region and 14 in IRB. All sequenced cpDNAs have been reported to contain SSRs and variation is said to occur within the species. This is why these sequences make good genetic markers in population studies and to estimate the relationships between plant taxa (Provan et al., 2001; Grassi et al., 2002; Melotto-Passarin et al., 2011).

### Phylogenomic Studies

A comparison of the *P. edulis* cp genome to those of the 22 species representing the four families of the Malpighiales order showed that each of the families (Passifloraceae, Salicaceae, Euphorbiaceae, and Chrysobalanaceae) constitutes a monophyletic group (Supplementary Table S1). The multiple-plastome alignment of the complete cp genome sequence was 208,695 bp in length. Removal of alignment columns containing gaps reduced the alignment length to 118,724 bp (nucleotide positions).

**FIGURE 5:**
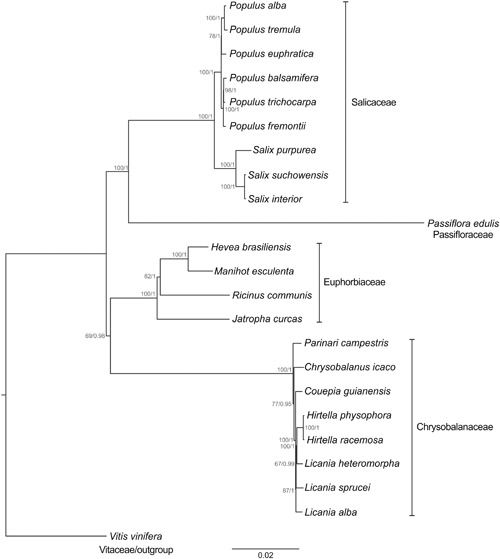
**Phylogenetic tree of the order Malpighiales inferred from the complete nucleotide sequence of the chloroplast genome.** Maximum likelihood analysis was applied to the TVM + G model, whereas BI was applied the GTR + G model. The bootstrap values for maximum likelihood and posterior probability for BI are indicated above each node. *Vitis vinifera* was used as outgroup to produce a rooted tree. The scale bar indicates the number of nucleotide substitutions per site.

Both maximum likelihod and BI-based methods resulted in a similar tree (**Figure 5**), and all species were clustered in Malpighiales, in conformity with the APG II system. The nodes resulting from ML analysis were consistently supported in the tree. The bootstrap values at most of the nodes (15 of 20 nodes) were ≥85%. In the BI the value of the average standard deviation of split frequencies was 0.0001 after 5,000,000 generations. The PSRF values were close to 1.0 (ranging between 0.999 and 1.000), and the ESS values were above 200 (ranging between 4,909 and 29,555) for all the parameters. All these values indicated that the analysis has reached convergence. The nodes resulting from BI analysis were also highly supported (PP = 1 for 17 nodes). *P. edulis* has been placed near the Salicaceae (*Salix* and *Populus* species) with strong support (BS = 100%; PP = 1), but distant from members of the Chrysobalanaceae, that in turn are closer to the Euphorbiaceae members studied herein.

In Malpighiales, a phylogeny based on plastid, mitochondrial and nuclear gene regions (Wurdack and Davis, 2009) and a recent phylogenomic approach based on a set of 82 chloroplast genes (Xi et al., 2012) indicated the relationship between Passifloraceae and Salicaceae. According to Wurdack and Davis (2009), for instance, most of its members share parietal placentation. Our results confirm this association and the potential of complete chloroplast genome sequences to infer evolutionary relationships. Malpighiales is an order that underwent rapid basal radiation. Therefore the use of large molecular data sets to identify several phylogenetically informative sites is an important step in deducing its course of evolution.

The Salicaceae species were clustered into two clades, both highly supported (BS = 100%; PP = 1). The most related were *S. purpurea* and *S. suchowensis* (BS = 100%; PP = 1), but positioned at some distance from those species is *S. interior* (BS = 100%, PP = 1). *Populus* species were split into two groups, the first incorporating *Populus alba*, *P. tremula*, and *P. euphratica* (BS = 78%; PP = 1), and the second *P. balsamifera*, *P. trichocarpa*, and *P. fremontii* (BS = 100%; PP = 1). The placement of *Salix* and *Populus* species is similar to that recently shown in the phylogenetic tree built from the cp DNA sequences of seven Salicaceae species (Wu, 2015).

With reference to Euphorbiaceae, *M. esculenta* was placed near to *H. brasiliensis* (BS = 100%; PP = 1), similar to the findings published by Xi et al. (2012) after examining a set of 82 chloroplast genes. Our findings placed *J. curcas* as the species most distant from the other Euphorbiaceae (BS = 100%; PP = 1), occupying an onward position relative to *R. communis* (BS = 82%; PP = 1). The placement of these species has weak support in two previous topologies based on 62 (Su et al., 2014) and 60 chloroplast genes (Kong and Yang, 2016). Remarkably, our study resolved the node positions in the Euphorbiaceae with strong support in both analyses (ML and BI). These findings also demonstrate the significance of using complete cp genomes in phylogeny reconstructions, since not only the genes but also non-coding sequences are examined. These sequences are informative, particularly at low taxonomic levels, due to their rapid evolution. In contrast, protein-coding genes evolve at a relatively slow rate (Folk et al., 2015).

Regarding the Chrysobalanaceae, the placement of *Licania heteromorpha* near to the species of *Hirtella* with strong support (BS = 100%; PP = 1) makes this genus paraphyletic. *Parinari campestris* was the most basal species in this family and this position is consistent with the findings of Bardon et al. (2013), based on six chloroplast markers and one nuclear marker. Moreover, the placement of the eight Chrysobalanaceae species with strong support is consistent with the phylogenetic hypothesis proposed by Malé et al. (2014) analyzing the complete plastid DNAs of eight species of this tropical family.

It is important to emphasize that our phylogenomic study is the first to take into account all the complete chloroplast genomes of the Malpighiales taxa available in databanks. Malpighiales includes 40 families, which are poorly represented in sequence databanks. Consequently, a limited taxonomic sampling of the order was used in the analysis and this may have contributed to the high support of the nodes in the phylogenetic trees obtained. Therefore, it is not possible to reach any definitive conclusions concerning the monophyly or position of the families within the order without examining the chloroplast genome sequences of members of the other families. However, our study lends support not only to the utility of complete chloroplast genomes to infer phylogenetic relationships, but as well as the enormous utility of long sequence reads from PacBio sequencing and assembly for generating very highly accurate biological information.

Subsequently, the complete nucleotide sequences of 43 chloroplast protein-coding genes of 42 species (including *P. edulis*) that belong to the eight orders of the Fabids clade were compared to reconstruct the phylogenomic relationships based on the GTR +G model (Supplementary Table S1), with *V. vinifera* (Vitales: Rosidae) as the outgroup. After alignment filtering and concatenating the nucleotide sequences into a single matrix, a total of 31,193 nucleotide positions were analyzed. Conservation analysis of positions was then performed and it is estimated that 61.65% correspond to conserved sites and 38.35% to variable sites. To summarize, these rates were estimated in Mega, a program that identifies a site as constant only if at least two sequences contain unambiguous nucleotides. In contrast, a variable site contains at least two types of nucleotide.

**FIGURE 6:**
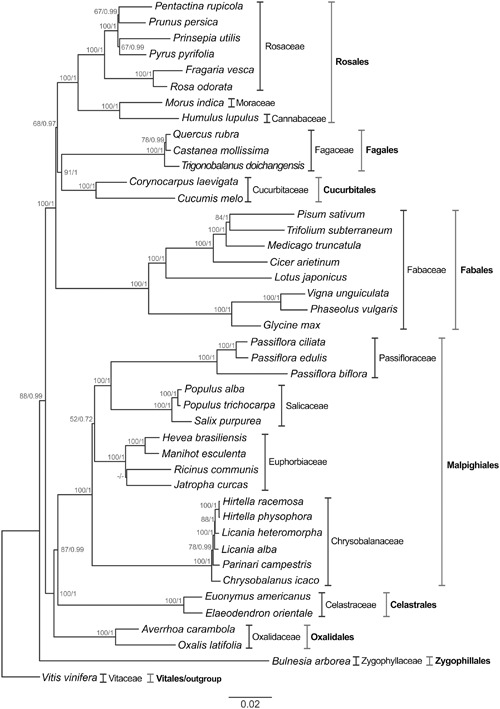
**Phylogenetic tree of the Fabids clade inferred from the nucleotide sequences of cp protein-coding genes of 43 plant species.** Both maximum likelihood and BI were applied to the GTR + G model. The bootstrap values for maximum likelihood and posterior probability for BI are indicated above each node; a dash indicates that the support nodes obtained were <50% or <0.50. *V. vinifera* was used as outgroup to produce a rooted tree. The scale bar indicates the number of nucleotide substitutions per site. Protein-coding genes used in the analysis: *atpA, atpB, atpE, atpH, atpI, ccsA, cemA, matK, ndhC, ndhE, ndhF, ndhG, ndhH, ndhI, ndhJ, ndhK, petA, petG, petL, petN, psaA, psaB, psaC, psaJ, psbA, psbC, psbD, psbE, psbF, psbI, psbJ, psbK, rbcL, rpl14, rpl36, rpoB, rpoC2, rps11, rps14, rps2, rps3, rps4, rps8*.

The method of analysis (ML or BI) had no substantial effect on the resulting trees. Their topologies were found to be highly similar (**Figure 6**). The accuracy of the inferred species’ phylogeny is strongly supported by the stability of the main clades obtained using different phylogenetic methods (ML and BI), and the best-scoring phylogenomic tree is shown in **Figure 6**. The nodes resulting from ML analysis were consistently supported in the tree (41 nodes in total). The bootstrap values at most of the nodes were higher than 85% (34 of 41), reaching 100% in some cases (28 nodes). In the BI the value of the average standard deviation of split frequencies was 0.0003 after 5,000,000 generations. The PSRF values were close to 1.0 (range between 0.999 and 1.000), and the ESS values were above 200 (range between 2,564 and 23,705) for all the parameters. All these values indicate that the analysis has reached convergence. The nodes resulting from BI analysis were also well-supported (PP = 1 for 31 nodes).

The Fabids consist of eight orders, which are all represented in this study and have been found to be monophyletic. For instance, *Bulnesia arborea* was the most distant species of the ingroup, thus placing the order Zygophyllales basal to the Fabids clade. This position of Zygophyllales has been previously reported (Wang et al., 2009).

Moreover, we were able to recognize two major monophyletic subgroups with strong support (BS = 100%; PP = 1). The first subgroup includes the orders Rosales, Fagales, Cucurbitales, and Fabales, known as the nitrogen-fixing clade, and the second consists of the orders Malpighiales, Celastrales, Oxalidales known as the COM clade. There is a trend in the literature toward classifying these subgroups as monophyletic (Wang et al., 2009; Su et al., 2014; Kong and Yang, 2016). Although there is morphological divergence among the species comprising the nitrogen-fixing clade, this capacity is shared only by angiosperms that belong to the orders Rosales, Fagales, Cucurbitales, and Fabales (Svistoonoff et al., 2013).

The members of the order Oxalidales (*Averrhoa carambola* and *Oxalis latifolia*) were placed as sister to a grade comprising the members of the order Celastrales (*Elaeodendron orientale* and *Euonymus americanus*) with strong support (BS = 100%; PP = 1). Thus, our studies indicate that Malpighiales shares more homologies with Celastrales than with Oxalidales, based on both methods used to infer species relationships within the Fabids. Previously, Hilu et al. (2003) reported a feasible relationship among the orders of the COM clade, based on sequence alignments of *matK*, a cp gene, but with weak support using maximum parsimony (BS = 60%). According to Sun et al. (2015), positioning the orders within the COM clade remains a great challenge. However, our results confirm other findings, for instance placing Celastrales nearer to Malpighiales than Oxalidales (Zhang and Simmons, 2006; Bell et al., 2010). The same scenario was described by Matthews and Endress (2005) observing floral structures and describing their implications for systematics in Celastrales. The use of a large set of genes is important for defining genomic relationships; otherwise the results could be interpreted as relating to the evolutionary course of particular genes.

It is worth noting that the Passifloraceae species comprise a distinct monophyletic group. In morphological terms, the monophyly of this family is supported by the occurrence of a flower corona (Judd et al., 2008). Additionally, our data suggest that *P. edulis* and *P. ciliata* are in close proximity. Both belong to the subgenus *Passiflora*. In taxonomic terms *P. edulis* is included in the supersection *Passiflora*, section *Passiflora*, and series *Passiflora*; *P. ciliate* is in the supersection *Stipulata*, section *Dysosmia*. However, *P. biflora* belongs to the subgenus Decaloba in supersection *Decaloba*, section *Decaloba* (Ulmer and MacDougal, 2004; Hansen et al., 2006). Passifloraceae appeared as a sister group to Salicaceae (BS = 100%; PP = 1) with a distant relationship to Chrysobalanaceae (BS = 52%; PP = 0.72) compared to Euphorbiaceae, confirming other findings based on four chloroplast, six mitochondrial and three nuclear gene sequences (Wurdack and Davis, 2009) and 82 chloroplast genes shared by 58 Malpighiales species (Xi et al., 2012).

However, we were unable to resolve the relationship between *J. curcas* and *R. communis* (BS ≤ 50%; PP = ≤ 0.50) in our work, confirming previous studies based on 60 chloroplast genes and the maximum likelihood method for estimating phylogeny inferences (Kong and Yang, 2016). Although the current phylogenetic reconstruction of Malpighiales is much improved compared to earlier versions, it is still incomplete, because of the limited taxonomic sampling available, and further phylogenetic and morphological studies are needed, focused especially on relations within Euphorbiaceae, by conducting, for instance, a phylogenomic analysis based on entire chloroplast genome sequences.

## Conclusion

In this study, it was possible to obtain the first complete sequence of a chloroplast genome for the Passifloraceae family using the SMRT sequencing method, which proved highly effective for generating the biological data and efficient in assembling the chloroplast genome of *P. edulis*. Chloroplast genomes of the order Malpighiales were compared, and although found to be highly conserved, some genomes such as *P. edulis* have undergone rearrangements during evolution, showing that there is diversity in the structure of plastid genomes in angiosperms. Definitely, complete chloroplast genomes or a significant set of their genic sequences are clearly useful in phylogenic studies. Additionally, our results have opened the way for future phylogenomic studies on Passifloraceae.

## Author Contributions

LC-S and CFM obtained the complete chloroplast (cp) genome from the BAC sequences, performing all the bioinformatic analyses and drafting the manuscript. LC-S was also responsible for the phylogenomic studies. NR and SC performed the BACDNA extraction and assembly of the PacBio sequences, assisted by HB at CNRGV, France. AAS analyzed the BAC-end sequences and identified clones containing cpDNA using the BAC library constructed by HAP. AV assisted with genome annotation and GCXO with the phylogenomic studies, contributing to the discussion of results, together with MCD. MLV conceived the study and drafted the final manuscript, which was read and approved by all authors.

## Conflict of Interest Statement

The authors declare that the research was conducted in the absence of any commercial or financial relationships that could be construed as a potential conflict of interest. The reviewer FTB and handling Editor declared their shared affiliation, and the handling Editor states that the process nevertheless met the standards of a fair and objective review.

## Footnotes

1 http://cnrgv.toulouse.inra.fr/Library/Passiflora

2 http://www.pacificbiosciences.com/

3 http://cnrgv.toulouse.inra.fr/fr/library/genomic_resource/Ped-B-Flav

4 http://www.gatc-biotech.com

5 http://pgrc.ipk-gatersleben.de/misa/

## Abbreviations

AIC: Akaike information criterion
BAC: bacterial artificial chromosome
BES: BAC-end sequencing
BI: Bayesian inference
bp: base pair
cp genome: chloroplast genome
GTR: general time reversible model
IR: inverted repeat
IRA: inverted repeat A
IRB: inverted repeat B
LSC: large single copy
ML: maximum likelihood
NGS: next generation sequencing
SSC: small single copy
SSR: simple sequence repeat
TVM: transversional model.
