# Supplementary material for: The Chloroplast Genome of Passiflora edulis (Passifloraceae) Assembled from Long Sequence Reads: Structural Organization and Phylogenomic Studies in Malpighiales
Source: Front Plant Sci. 2017 Mar 10;8:334. doi: 10.3389/fpls.2017.00334 (PMC5345083; doi:10.3389/fpls.2017.00334)
Supplement: Supplementary file 2 [file Image_1.PDF]

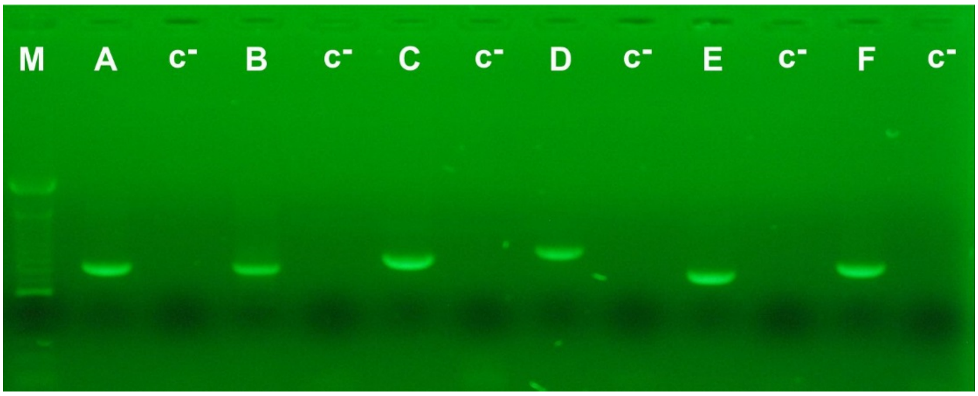

Supplementary Figure S1. LSC inversions validated by Sanger sequencing**.** 1a. PCR products run on an agarose gel (1 %) using primers to anneal inversion boundaries. **A** – inversion 1, 5'end; **B** – inversion 1, 3'end; **C** – inversion 2, 5'end; **D** – inversion 2, 3'end; **E** – inversion 3, 5'end; **F** – inversion 3, 3'end. **c**$^{-}$ – PCR assay without DNA template as a negative control. M, 100-bp ladder DNA molecular size marker (Invitrogen®). Primer annealing sites are shown below (1b – 1g).

```
inv1_5'end_PacBio    1 AGAGGGTTCACGCAGAAGAGATATAGATATTTTTTTCTAAGAGGTTGTT    50
                                        |||||||||||||||||||||||||||||||||
inv1_5'end_Sanger    1 -----------------GAGATATAGATATTTTTTTCTAAGAGGTTGTT    33

inv1_5'end_PacBio   51 ATAATATGATTGAAGCGCGGAAGAGGGCTTTATGAAACATACACAACACA   100
                       ||||||||||||||||||||||||||||||||||||||||||||||||||
inv1_5'end_Sanger   34 ATAATATGATTGAAGCGCGGAAGAGGGCTTTATGAAACATACACAACACA    84

inv1_5'end_PacBio  101 GATAAAAGGATAGTTATTTATATATATGTCTATGTCTCCCCTGTTAGTGC   150
                       ||||||||||||||||||||||||||||||||||||||||||||||||||
inv1_5'end_Sanger   85 GATAAAAGGATAGTTATTTATATATATGTCTATGTCTCCCCTGTTAGTGC   135

inv1_5'end_PacBio  151 AGTTCGATTCTGGACAGCGTCGTGCTCTGGCAAAACCTCATAGAAAATCG   200
                       ||||||||||||||||||||||||||||||||||||||||||||||||||
inv1_5'end_Sanger  136 AGTTCGATTCTGGACAGCGTCGTGCTCTGGCAAAACCTCATAGAAAATCG   186

inv1_5'end_PacBio  201 ATTCTATGAGTTATAGAAATATAAGATTTCCGATTAGATATTCGCATAAG   250
                       ||||||||||||||||||||||||||||||||||||||||||||||||||
inv1_5'end_Sanger  187 ATTCTATGAGTTATAGAAATATAAGATTTCCGATTAGATATTCGCATAAG   237

inv1_5'end_PacBio  251 AATTTGGGTTCTGGGGTTGACATATATATAGATTCTATGTTAGAATAGAA   300
                       ||||||||||||||||||||||||||||||||||||||||||||||||||
inv1_5'end_Sanger  238 AATTTGGGTTCTGGGGTTGACATATATATAGATTCTATGTTAGAATAGAA   288

inv1_5'end_PacBio  301 TCTGAAAATAGAGAAGAGCAAAATTTCGGTTCTGTCTGGGGTTGACATAT   350
                       ||||||||||||||||||||||||||||||||||||||||||||||||||
inv1_5'end_Sanger  289 TCTGAAAATAGAGAAGAGCAAAATTTCGGTTCTGTCTGGGGTTGACATAT   339

inv1_5'end_PacBio  351 TTGTCTACCAAAAAGACAATAAATTCATTTTTATGAGGATACCTCTCTAT   400
                       ||||||||||||||||||||||||||||||||||||||||||||||||||
inv1_5'end_Sanger  340 TTGTCTACCAAAAAGACAATAAATTCATTTTTATGAGGATACCTCTCTAT   390

inv1_5'end_PacBio  401 TAAAGGAAACTCGAATACTTAATGCTTTATGCTTTACATTATAAAAAATG   450
                       ||||||||||||||||||||||||||||||||||||||||||||||||||
inv1_5'end_Sanger  391 TAAAGGAAACTCGAATACTTAATGCTTTATGCTTTACATTATAAAAAATG   441

inv1_5'end_PacBio  451 CCCCTTGGTGTTCCAAAAGTACCCTTTGACTTTGACGTTGACTTTGACCG   500
                       ||||||||||||||||||||||||||||||||||||||||||||||||||
inv1_5'end_Sanger  442 CCCCTTGGTGTTCCAAAAGTACCCTTTGACTTTGACGTTGACTTTGACCG   492
```

```
inv1_5’end_PacBio 501 TCCCGAATACGAATACCGCGAAGAAGACGAAGAGTCAACTTGGGTTGACT     550
                      ||||||||||||||||||||||||||||||||||||||||||||||||||
inv1_5’end_Sanger 493 TCCCGAATACGAATACCGCGAAGAAGACGAAGAGTCAACTTGGGTTGACT     543

inv1_5’end_PacBio 551 TATACAATGCACTTTATAAAAAGAGACAGTTGTTTTTGTTTCAAGACGTT     600
                      ||||||||||||||||||||||||||||||||||||||||||||||||||
inv1_5’end_Sanger 544 TATACAATGCACTTTATAAAAAGAGACAGTTGTTTTTGTTTCAAGACGTT     594

inv1_5’end_PacBio 601 AATAGCGAGATAGCGAATCAACTTATCAACCTTTTTCTATATCTCGATTC     650
                      ||||||||||||||||||||||||||||||||||||||||||||||||||
inv1_5’end_Sanger 595 AATAGCGAGATAGCGAATCAACTTATCAACCTTTTTCTATATCTCGATTC     645

inv1_5’end_PacBio 651 AGAAAATGATATCACAGATTTTTGTCTTTATATAGACTCTCCCGGGGGAT     700
                      ||||||||||||||||||||||||||||||||||||||||||||||||||
inv1_5’end_Sanger 646 AGAAAATGATATCACAGATTTTTGTCTTTATATAGACTCTCCCGGGGGAT     696

inv1_5’end_PacBio 701 GGATACTCCCAGGTATTAGTCTTTATAATGTTATGGAGGCTGTGGAGTCG     750
                      ||||||||||||||||||||||||||||||||||||||||||||||||||
inv1_5’end_Sanger 697 GGATACTCCCAGGTATTAGTCTTTATAATGTTATGGAGGCTGTGGAGTCG     747

inv1_5’end_PacBio 751 GATATACGAACAATATGCGTCGGACTGGCCGCTTCAATGGCATCTTTTAT     800
                      ||||||||||||||||||||||||||||||||||||||||||||||||||
inv1_5’end_Sanger 748 GATATACGAACAATATGCGTCGGACTGGCCGCTTCAATGGCATCTTTTAT     798

inv1_5’end_PacBio 801 CCTGGCCGTGGGAAATCCTAACAAACGTGTAGCATTCCCTCACGCT         846
                      |||||||||||||||||||||||||||||
inv1_5’end_Sanger 799 CCTGGCCGTGGGAAATCCTAACAAACGTG-----------------         827
```

1b. Alignment of the PacBio sequence (in red) with the respective amplicon sequence obtained by the Sanger method (in dark blue). The flanking region and the beginning of inversion 1 (in bold) are shown. Nucleotides highlighted in yellow indicate primer annealing sites.

```
inv1_3’end_PacBio   1 ACTGGGGAACGAGGATTTGCAGTCCTTCGCCTTACCACTCGGCCATGCCG      50
                                  ||||||||||||||||||||||||||||||||||||||
inv1_3’end_Sanger   1 ------------GGATTTGCAGTCCTTCGCCTTACCACTCGGCCATGCCG      38

inv1_3’end_PacBio  51 CCCCAACTAAGGGGATTTCCAATGTTGATTGGTCCCCAACCAACATTTAA     100
                      ||||||||||||||||||||||||||||||||||||||||||||||||||
inv1_3’end_Sanger  39 CCCCAACTAAGGGGATTTCCAATGTTGATTGGTCCCCAACCAACATTTAA      89

inv1_3’end_PacBio 101 CTATCGACTGTAAAGAGGATTTGGAGTCCTCTTTACAACCATTTAACTAC     150
                      ||||||||||||||||||||||||||||||||||||||||||||||||||
inv1_3’end_Sanger  90 CTATCGACTGTAAAGAGGATTTGGAGTCCTCTTTACAACCATTTAACTAC     140

inv1_3’end_PacBio 151 AACCATTTAACTACCGACAGAGGATTTGGAGTCCTCTTTACAACCATTTA     200
                      ||||||||||||||||||||||||||||||||||||||||||||||||||
inv1_3’end_Sanger 141 AACCATTTAACTACCGACAGAGGATTTGGAGTCCTCTTTACAACCATTTA     191

inv1_3’end_PacBio 201 ACTACAACCATTTAACTACCGACAGAGGATTTGGAGTCCCCAACCGACGA     250
                      ||||||||||||||||||||||||||||||||||||||||||||||||||
inv1_3’end_Sanger 192 ACTACAACCATTTAACTACCGACAGAGGATTTGGAGTCCCCAACCGACGA     242

inv1_3’end_PacBio 251 CTGTAAAGGGGATTTCCAGTCCCCCAGTCGCCAACCAACATTTAACTACC     300
                      ||||||||||||||||||||||||||||||||||||||||||||||||||
inv1_3’end_Sanger 243 CTGTAAAGGGGATTTCCAGTCCCCCAGTCGCCAACCAACATTTAACTACC     293

inv1_3’end_PacBio 301 GAAGAGAAAGGGGATTTGGAGTCCCCAACCAACATTAACCACCATTTAAC     350
                      ||||||||||||||||||||||||||||||||||||||||||||||||||
inv1_3’end_Sanger 294 GAAGAGAAAGGGGATTTGGAGTCCCCAACCAACATTAACCACCATTTAAC     344
```

```
inv1_3’end_PacBio 351 TATCGACTGTGAATTCATGAATCATGAACCATTCTTAGATTTTAATCTAA    400
                      ||||||||||||||||||||||||||||||||||||||||||||||||||
inv1_3’end_Sanger 345 TATCGACTGTGAATTCATGAATCATGAACCATTCTTAGATTTTAATCTAA    395

inv1_3’end_PacBio 401 CGTTGCATTTCCTTAAAAATATAGGAAAATAAAAAAGGATATGTAACTAT    450
                      ||||||||||||||||||||||||||||||||||||||||||||||||||
inv1_3’end_Sanger 396 CGTTGCATTTCCTTAAAAATATAGGAAAATAAAAAAGGATATGTAACTAT    446

inv1_3’end_PacBio 451 TTAGTATTGATTCTAAAAAACCAATCAATTGTTCTCTTCTCCATTTTCAT    500
                      ||||||||||||||||||||||||||||||||||||||||||||||||||
inv1_3’end_Sanger 447 TTAGTATTGATTCTAAAAAACCAATCAATTGTTCTCTTCTCCATTTTCAT    497

inv1_3’end_PacBio 501 ATTCTATATATAATATAGAATCTATCTATATGTCAACCCCTTCTTTCTTC    550
                      ||||||||||||||||||||||||||||||||||||||||||||||||||
inv1_3’end_Sanger 498 ATTCTATATATAATATAGAATCTATCTATATGTCAACCCCTTCTTTCTTC    548

inv1_3’end_PacBio 551 TCCATTTTCATATTCTATATATAATATAGAATCTATCTATATGTCAACCC    600
                      ||||||||||||||||||||||||||||||||||||||||||||||||||
inv1_3’end_Sanger 549 TCCATTTTCATATTCTATATATAATATAGAATCTATCTATATGTCAACCC    599

inv1_3’end_PacBio 601 CTTCTTTCTTCTCCATTTTCATATTCTATATATAATATAGAATCTATCTA    650
                      ||||||||||||||||||||||||||||||||||||||||||||||||||
inv1_3’end_Sanger 600 CTTCTTTCTTCTCCATTTTCATATTCTATATATAATATAGAATCTATCTA    650

inv1_3’end_PacBio 651 TATGTCAACCCCTTCTTTCTTCTCCATTTTCATATTCTATATATAATATA    700
                      ||||||||||||||||||||||||||||||||||||||||||||||||||
inv1_3’end_Sanger 651 TATGTCAACCCCTTCTTTCTTCTCCATTTTCATATTCTATATATAATATA    701

inv1_3’end_PacBio 701 GAATCTATCTATATGTCAACCCCAGCTAGAACAGATATAGAATATAAAAG    750
                      ||||||||||||||||||||||||||||||||||||||||||||||||||
inv1_3’end_Sanger 702 GAATCTATCTATATGTCAACCCCAGCTAGAACAGATATAGAATATAAAAG    752

inv1_3’end_PacBio 751 TATCCTCATAAAAATGAATTTATTGTCTTAATAATACCGGTCCTTATCCC    800
                      ||||||||||||||||||||||||||||||||||||||||||||||||||
inv1_3’end_Sanger 753 TATCCTCATAAAAATGAATTTATTGTCTTAATAATACCGGTCCTTATCCC    803

inv1_3’end_PacBio 801 ATTTTATGCGCAGATTAGATAAGTTCAGTGG  831
                      |||||||||||||
inv1_3’end_Sanger 804 ATTTTATGCGCAG------------------  816
```

1c. Alignment of the PacBio sequence (in red) with the respective amplicon sequence obtained by the Sanger method (in dark blue). The end of inversion 1 (in bold) and the flanking region are shown. Nucleotides highlighted in yellow indicate primer annealing sites.

```
inv2_5’end_PacBio   1 ATCGGTCCACACGGTTGTCCATGTACCAGTAGAAGATTCAGCAGCTACTG     50
                                         |||||||||||||||||||||||||||||||
inv2_5’end_Sanger   1 -------------------CATGTACCAGTAGAAGATTCAGCAGCTACTG     31

inv2_5’end_PacBio  51 CAGCTCCCGCTTCCTCAGCGGGAACTCCAGGTTGAGGAGTTACTCGGAAT    100
                      ||||||||||||||||||||||||||||||||||||||||||||||||||
inv2_5’end_Sanger  32 CAGCTCCCGCTTCCTCAGCGGGAACTCCAGGTTGAGGAGTTACTCGGAAT     82

inv2_5’end_PacBio 101 GCTGCCAAGATATCAGTATCTTTGGGGTTATATTCAGGAGTATAATAAGT    150
                      ||||||||||||||||||||||||||||||||||||||||||||||||||
inv2_5’end_Sanger  83 GCTGCCAAGATATCAGTATCTTTGGGGTTATATTCAGGAGTATAATAAGT    133

inv2_5’end_PacBio 151 CAATTTATAATCTTTAACACCAGCCTTGAATCCAACACTTGCTTTAGTCT    200
                      ||||||||||||||||||||||||||||||||||||||||||||||||||
inv2_5’end_Sanger 134 CAATTTATAATCTTTAACACCAGCCTTGAATCCAACACTTGCTTTAGTCT    184
```

```
inv2_5’end_PacBio 201 CTGTTTGTGGTGACATAAGTCCCTCCCTACAACTCATGAATTCAAAATTC    250
                      ||||||||||||||||||||||||||||||||||||||||||||||||||
inv2_5’end_Sanger 185 CTGTTTGTGGTGACATAAGTCCCTCCCTACAACTCATGAATTCAAAATTC    235

inv2_5’end_PacBio 251 TCACAGCAACAAGGTCTACTCGAGATGAATTCGAATTAGGAGTTAATGAA    300
                      ||||||||||||||||||||||||||||||||||||||||||||||||||
inv2_5’end_Sanger 176 TCACAGCAACAAGGTCTACTCGAGATGAATTCGAATTAGGAGTTAATGAA    286

inv2_5’end_PacBio 301 ACCTTTCACAGGAATCCTTCACAAAGCTATCAATCAATATTCTCAACTAA    350
                      ||||||||||||||||||||||||||||||||||||||||||||||||||
inv2_5’end_Sanger 287 ACCTTTCACAGGAATCCTTCACAAAGCTATCAATCAATATTCTCAACTAA    337

inv2_5’end_PacBio 351 TCAGAATTTCATTATTTTTCCATATTATTTGATTCACCAAATACATCATT    400
                      ||||||||||||||||||||||||||||||||||||||||||||||||||
inv2_5’end_Sanger 338 TCAGAATTTCATTATTTTTCCATATTATTTGATTCACCAAATACATCATT    388

inv2_5’end_PacBio 401 ATTGTATACTCTTTCATATATATGATGCAACCTCATCCTTGTTTTTTCAA    450
                      ||||||||||||||||||||||||||||||||||||||||||||||||||
inv2_5’end_Sanger 389 ATTGTATACTCTTTCATATATATGATGCAACCTCATCCTTGTTTTTTCAA    439

inv2_5’end_PacBio 451 GTTTCTAATCCTTAACCTTTTTATTTCTATTTCAGCTTTAGGCTCTTCT    500
                      ||||||||||||||||||||||||||||||||||||||||||||||||||
inv2_5’end_Sanger 440 GTTTCTAATCCTTAACCTTTTTATTTCTATTTCAGCTTTAGGCTCTTCT    490

inv2_5’end_PacBio 501 CTGAATGATTCTTGTGAGCAAGCTTATGAGAATGATTTTTTTTCGATGAA    550
                      ||||||||||||||||||||||||||||||||||||||||||||||||||
inv2_5’end_Sanger 491 CTGAATGATTCTTGTGAGCAAGCTTATGAGAATGATTTTTTTTCGATGAA    541

inv2_5’end_PacBio 551 CCAAGGAATCCCAAGTGGCCAGTTACAAACAATACAATAATGAAGCAATA    600
                      ||||||||||||||||||||||||||||||||||||||||||||||||||
inv2_5’end_Sanger 452 CCAAGGAATCCCAAGTGGCCAGTTACAAACAATACAATAATGAAGCAATA    592

inv2_5’end_PacBio 601 AAAACTATACAATTTTTTTAGGTAGGGCTATACGGACTCGAACCGTAGAC    650
                      ||||||||||||||||||||||||||||||||||||||||||||||||||
inv2_5’end_Sanger 593 AAAACTATACAATTTTTTTAGGTAGGGCTATACGGACTCGAACCGTAGAC    643

inv2_5’end_PacBio 651 TTTCTCGGTAAAACAGATTGAACTGATTATTATCAAAAAGATTCGAACTG    700
                      ||||||||||||||||||||||||||||||||||||||||||||||||||
inv2_5’end_Sanger 644 TTTCTCGGTAAAACAGATTGAACTGATTATTATCAAAAAGATTCGAACTG    694

inv2_5’end_PacBio 701 TTTCAAAGACCCAACATGCACTTTTTTGCATTGGGCTCTTTCATTAACTG    750
                      ||||||||||||||||||||||||||||||||||||||||||||||||||
inv2_5’end_Sanger 695 TTTCAAAGACCCAACATGCACTTTTTTGCATTGGGCTCTTTCATTAACTG    745

inv2_5’end_PacBio 751 ATAGAAAGAATCAGTTAGTCTACCATAATTTTCTTGACAGAAAGATAACA    800
                      ||||||||||||||||||||||||||||||||||||||||||||||||||
inv2_5’end_Sanger 746 ATAGAAAGAATCAGTTAGTCTACCATAATTTTCTTGACAGAAAGATAACA    796

inv2_5’end_PacBio 801 AGATGGCTCCGCGTGCTCTGATTTATTATTTTGATTCCGATCTCAAAGCA    850
                      ||||||||||||||||||||||||||||||||||||||||||||||||||
inv2_5’end_Sanger 797 AGATGGCTCCGCGTGCTCTGATTTATTATTTTGATTCCGATCTCAAAGCA    847

inv2_5’end_PacBio 851 CTACCAAAGTGTTTCAAAGAAGGTTATGCTGACGTAGGTTTGCTTTTGGC    900
                      ||||||||||||||||||||||||||||||||||||||||||||||||||
inv2_5’end_Sanger 848 CTACCAAAGTGTTTCAAAGAAGGTTATGCTGACGTAGGTTTGCTTTTGGC    898

inv2_5’end_PacBio 901 TTAGATTAACCTAAGTTAAATGGAGTTTCCATCGCCCCGCTTTT      944
                      |||||||||||||||||||||||||||
inv2_5’end_Sanger 899 TTAGATTAACCTAAGTTAAATGGAGTT-----------------      925
```

1d. Alignment of the PacBio sequence (in red) with the respective amplicon sequence obtained by the Sanger method (in dark blue). The flanking region and the beginning of inversion 2 (in bold) are shown. Nucleotides highlighted in yellow indicate primer annealing sites.

```
inv2_3'end_PacBio    1  GAAGCAGTAGGATGGATTTTCATAATAATGGAAACTCTAAGACCAGAAGC   50
                                                                   |||
inv2_3'end_Sanger    1  -----------------------------------------------AGC    3

inv2_3'end_PacBio   51  AGTAAGATTGATTCTCATAATCATAATAATATAATAATAAATAAAATATG  100
                        ||||||||||||||||||||||||||||||||||||||||||||||||||
inv2_3'end_Sanger    4  AGTAAGATTGATTCTCATAATCATAATAATATAATAATAAATAAAATATG   54

inv2_3'end_PacBio  101  TCGAATTTTTTTTTTCGAAAATTTCGAGTCCAAAAGAAATCTTCAATAG  150
                        ||||||||||||||||||||||||||||||||||||||||||||||||||
inv2_3'end_Sanger   55  TCGAATTTTTTTTTTCGAAAATTTCGAGTCCAAAAGAAATCTTCAATAG  105

inv2_3'end_PacBio  151  CAAGTAACAAGTTGATCGATTAATTTAATAAGAAAAAGAAATGGGAGTTA  200
                        ||||||||||||||||||||||||||||||||||||||||||||||||||
inv2_3'end_Sanger  106  CAAGTAACAAGTTGATCGATTAATTTAATAAGAAAAAGAAATGGGAGTTA  156

inv2_3'end_PacBio  201  GTGCTTGATTTGATTGGTATCATTCAAGGACTCAAATTCCATTGTTTACT  250
                        ||||||||||||||||||||||||||||||||||||||||||||||||||
inv2_3'end_Sanger  157  GTGCTTGATTTGATTGGTATCATTCAAGGACTCAAATTCCATTGTTTACT  207

inv2_3'end_PacBio  251  TATTTTCAATTTCAATGAGTGAATTTTCAAGTTCGACTAACTCATTTTCA  300
                        ||||||||||||||||||||||||||||||||||||||||||||||||||
inv2_3'end_Sanger  208  TATTTTCAATTTCAATGAGTGAATTTTCAAGTTCGACTAACTCATTTTCA  258

inv2_3'end_PacBio  301  CCAAATGGATGAATAAAAAAAAGCTTCAGGAAGCCTTTCATTTGTCTATC  350
                        ||||||||||||||||||||||||||||||||||||||||||||||||||
inv2_3'end_Sanger  259  CCAAATGGATGAATAAAAAAAAGCTTCAGGAAGCCTTTCATTTGTCTATC  309

inv2_3'end_PacBio  351  ATTATAGACAATACTATCCATATTATCTATGGAATTCAAACCTGAACTCG  400
                        ||||||||||||||||||||||||||||||||||||||||||||||||||
inv2_3'end_Sanger  310  ATTATAGACAATACTATCCATATTATCTATGGAATTCAAACCTGAACTCG  361

inv2_3'end_PacBio  401  ATTTTCTATTTAATTTATTTTATTCTATTCAATTTTTGAATTTACGATTC  450
                        ||||||||||||||||||||||||||||||||||||||||||||||||||
inv2_3'end_Sanger  362  ATTTTCTATTTAATTTATTTTATTCTATTCAATTTTTGAATTTACGATTC  412

inv2_3'end_PacBio  451  ACTATTTCTATCTCATAGGCCTTTCATTTTGTCTTTCAACATATCGATTT  500
                        ||||||||||||||||||||||||||||||||||||||||||||||||||
inv2_3'end_Sanger  413  ACTATTTCTATCTCATAGGCCTTTCATTTTGTCTTTCAACATATCGATTT  463

inv2_3'end_PacBio  501  ACGCCCAGTTATTATTTCTGTTTTTTTTTGGGGGGGGTACTCGACTAAGC  550
                        ||||||||||||||||||||||||||||||||||||||||||||||||||
inv2_3'end_Sanger  464  ACGCCCAGTTATTATTTCTGTTTTTTTTTGGGGGGGGTACTCGACTAAGC  514

inv2_3'end_PacBio  551  CTAATGAATTTTTGGAGTCAAAATCATGACATGAATCCGGTTAAAAACAA  600
                        ||||||||||||||||||||||||||||||||||||||||||||||||||
inv2_3'end_Sanger  515  CTAATGAATTTTTGGAGTCAAAATCATGACATGAATCCGGTTAAAAACAA  565

inv2_3'end_PacBio  601  AATTCCAACTTAACCCAACCAAAACGGAATCCGCTACTAAATTACGTAGG  650
                        ||||||||||||||||||||||||||||||||||||||||||||||||||
inv2_3'end_Sanger  566  AATTCCAACTTAACCCAACCAAAACGGAATCCGCTACTAAATTACGTAGG  616

inv2_3'end_PacBio  651  GTTAATAACGACTTACTAAATTTATGCGCAAGCCATGTAGTTTGAAAGGA  700
                        ||||||||||||||||||||||||||||||||||||||||||||||||||
inv2_3'end_Sanger  617  GTTAATAACGACTTACTAAATTTATGCGCAAGCCATGTAGTTTGAAAGGA  667

inv2_3'end_PacBio  701  TCTTTGATGACTTTCCTTATTAACATTGTCAAATAGGCCTTTCTTAGTAT  750
                        ||||||||||||||||||||||||||||||||||||||||||||||||||
inv2_3'end_Sanger  668  TCTTTGATGACTTTCCTTATTAACATTGTCAAATAGGCCTTTCTTAGTAT  718

inv2_3'end_PacBio  751  ACCTTAACTGGTGAAGCACAAAATAAAGTAAGTAGTCTTTTCTTGCCCAA  800
                        ||||||||||||||||||||||||||||||||||||||||||||||||||
inv2_3'end_Sanger  719  ACCTTAACTGGTGAAGCACAAAATAAAGTAAGTAGTCTTTTCTTGCCCAA  769
```

```
inv2_3’end_PacBio 801 ATTTCATTAATATGAAAATTGAATTAATATTCAATTGAATAAGCTTCTAA    850
                      ||||||||||||||||||||||||||||||||||||||||||||||||||
inv2_3’end_Sanger 770 ATTTCATTAATATGAAAATTGAATTAATATTCAATTGAATAAGCTTCTAA    820

inv2_3’end_PacBio 851 TAATCTATTATGTATTCTAATTTCTTTATTCTAATAAAAAAAAATACTAA    900
                      ||||||||||||||||||||||||||||||||||||||||||||||||||
inv2_3’end_Sanger 821 TAATCTATTATGTATTCTAATTTCTTTATTCTAATAAAAAAAAATACTAA    871

inv2_3’end_PacBio 901 TAAAAAAAATTCGAAAAAAAAAAGACATTTGAATTTCTTTAATATTCAAT    950
                      ||||||||||||||||||||||||||||||||||||||||||||||||||
inv2_3’end_Sanger 872 TAAAAAAAATTCGAAAAAAAAAAGACATTTGAATTTCTTTAATATTCAAT    922

inv2_3’end_PacBio 951 TTCTAATAGAATTTAGAACTTCTAAACAAAAAATGAGAATTCTATTTTGA   1000
                      |||||||||||||||||||||||||||||||||
inv2_3’end_Sanger 923 TTCTAATAGAATTTAGAACTTCTAAACAAAAAA-----------------    955

inv2_3’end_PacBio 1001 ATTCCTTTTTGGTATAGAGAATCGAAAGCG   1030

inv2_3’end_Sanger      ------------------------------
```

1e. Alignment of the PacBio sequence (in red) with the respective amplicon sequence obtained by the Sanger method (in dark blue). The end of inversion 2 (in bold) and the flanking region are shown. Nucleotides highlighted in yellow indicate primer annealing sites.

```
inv3_5’end_PacBio   1 GGGCATCCACCATCATACCTACAATGATTGGCCATACTATCGCTATCCAT     50
                                        ||||||||||||||||||||||||||||||||
inv3_5’end_Sanger   1 ------------------CTACAATGATTGGCCATACTATCGCTATCCAT     32

inv3_5’end_PacBio  51 AATGGAAAAGAAAAAATACCTATTTATATAACAGATTATATGGTGGGTCA    100
                      ||||||||||||||||||||||||||||||||||||||||||||||||||
inv3_5’end_Sanger  33 AATGGAAAAGAAAAAATACCTATTTATATAACAGATTATATGGTGGGTCA     83

inv3_5’end_PacBio 101 TAAATTGGGAGAATTTTCACCTACTCTTTCTTTCAAGGGGCATCCAAAAA    150
                      ||||||||||||||||||||||||||||||||||||||||||||||||||
inv3_5’end_Sanger  84 TAAATTGGGAGAATTTTCACCTACTCTTTCTTTCAAGGGGCATCCAAAAA    134

inv3_5’end_PacBio 151 ATGATAATAAATCTCGTCGTTAGTTTGATTATTTTGAAACTTTTAAAAGT    200
                      ||||||||||||||||||||||||||||||||||||||||||||||||||
inv3_5’end_Sanger 135 ATGATAATAAATCTCGTCGTTAGTTTGATTATTTTGAAACTTTTAAAAGT    185

inv3_5’end_PacBio 201 GAAAATAAAAGTGAAAAGGAATTGGATAAAAGTGAAAAGGAATTGGAAAA    250
                      ||||||||||||||||||||||||||||||||||||||||||||||||||
inv3_5’end_Sanger 186 GAAAATAAAAGTGAAAAGGAATTGGATAAAAGTGAAAAGGAATTGGAAAA    236

inv3_5’end_PacBio 251 GTAATCGTTTTTTTACTAAATAGTTTTTTGACTTTTTTTATAGATATAGA    300
                      ||||||||||||||||||||||||||||||||||||||||||||||||||
inv3_5’end_Sanger 237 GTAATCGTTTTTTTACTAAATAGTTTTTTGACTTTTTTTATAGATATAGA    287

inv3_5’end_PacBio 301 TTCTTTTTTGAAATGAAAATGAATAGTAGAACAAAGAAGAAGAAGAAATC    350
                      ||||||||||||||||||||||||||||||||||||||||||||||||||
inv3_5’end_Sanger 288 TTCTTTTTTGAAATGAAAATGAATAGTAGAACAAAGAAGAAGAAGAAATC    338

inv3_5’end_PacBio 351 AAAATCCTATTTTTGAACTGATTTCCACTTTATTCATTCAAAAGAATTCT    400
                      ||||||||||||||||||||||||||||||||||||||||||||||||||
inv3_5’end_Sanger 339 AAAATCCTATTTTTGAACTGATTTCCACTTTATTCATTCAAAAGAATTCT    389

inv3_5’end_PacBio 401 AATGAATCGAGACGGATAAGATCATAAAGATGTATCTATGAATGTTGATC    450
                      ||||||||||||||||||||||||||||||||||||||||||||||||||
inv3_5’end_Sanger 390 AATGAATCGAGACGGATAAGATCATAAAGATGTATCTATGAATGTTGATC    440
```

```
inv3_5'end_PacBio 451 TTGGTTGACACGGGTATATGAGTCATGTTATACTGTTGAGTAACAAGCCC     500
                      ||||||||||||||||||||||||||||||||||||||||||||||||||
inv3_5'end_Sanger 441 TTGGTTGACACGGGTATATGAGTCATGTTATACTGTTGAGTAACAAGCCC     491

inv3_5'end_PacBio 501 CCAACTCTCTATTCTATTTTTTGTTCTAGAGAATTGGTGTACTTGGGAGT     550
                      ||||||||||||||||||||||||||||||||||||||||||||||||||
inv3_5'end_Sanger 492 CCAACTCTCTATTCTATTTTTTGTTCTAGAGAATTGGTGTACTTGGGAGT     542

inv3_5'end_PacBio 551 CCCTGATGATTAAATAAACCAAGATTTTACCATGACTGCAATTTTAGAGA     600
                      ||||||||||||||||||||||||||||||||||||||||||||||||||
inv3_5'end_Sanger 543 CCCTGATGATTAAATAAACCAAGATTTTACCATGACTGCAATTTTAGAGA     593

inv3_5'end_PacBio 601 GACGCGAAAGCGAAAGCCTATGGGGTCGTTTCTGTAACTGGATAACCAGC     650
                      ||||||||||||||||||||||||||||||||||||||||||||||||||
inv3_5'end_Sanger 594 GACGCGAAAGCGAAAGCCTATGGGGTCGTTTCTGTAACTGGATAACCAGC     644

inv3_5'end_PacBio 651 ACCGAAAACCGTCTTTACATTGGATGGTTTGGTGTTTTGATGATCCCTAC     700
                      ||||||||||||||||||||||||||||||||||||||||||||||||||
inv3_5'end_Sanger 645 ACCGAAAACCGTCTTTACATTGGATGGTTTGGTGTTTTGATGATCCCTAC     695

inv3_5'end_PacBio 701 TTTATTGACCGCAACTTCTGTATTTATTATCGCTTTCATTGCTGCCCCTC     750
                      ||||||||||||||||||||||||||||||||||||||||||||||||||
inv3_5'end_Sanger 696 TTTATTGACCGCAACTTCTGTATTTATTATCGCTTTCATTGCTGCCCCTC     746

inv3_5'end_PacBio 751 CAGTAGATATTGATGGTATTCGTGAACCCGTTTC     784
                      |||||||||||||||||||||
inv3_5'end_Sanger 747 CAGTAGATATTGATGGTATTC-------------     767
```

1f. Alignment of the PacBio sequence (in red) with the respective amplicon sequence obtained by the Sanger method (in dark blue). The flanking region and the beginning of inversion 3 (in bold) are shown. Nucleotides highlighted in yellow indicate primer annealing sites.

```
inv3_3'end_PacBio   1 ATCAACCGTGCTAACCTTGGTATGGAAGTTATGCATGAACGTAATGCTCA      50
                                         |||||||||||||||||||||||||||||||
inv3_3'end_Sanger   1 -------------------GTATGGAAGTTATGCATGAACGTAATGCTCA      31

inv3_3'end_PacBio  51 CAACTTCCCTCTAGACCTAGCTGCTGTTGAAGCTCCATCTACAAATGGGT     100
                      ||||||||||||||||||||||||||||||||||||||||||||||||||
inv3_3'end_Sanger  32 CAACTTCCCTCTAGACCTAGCTGCTGTTGAAGCTCCATCTACAAATGGGT      83

inv3_3'end_PacBio 101 AAGACTTTGGTCTTAGTGTGTACAAGTTCGTGAAATAAAGAAAGGAGCAA     150
                      ||||||||||||||||||||||||||||||||||||||||||||||||||
inv3_3'end_Sanger  84 AAGACTTTGGTCTTAGTGTGTACAAGTTCGTGAAATAAAGAAAGGAGCAA     134

inv3_3'end_PacBio 151 TAACAATCTTCTTGATATAACAAGAAATTGGCTATTGCTCCTTTCTTCAT     200
                      ||||||||||||||||||||||||||||||||||||||||||||||||||
inv3_3'end_Sanger 135 TAACAATCTTCTTGATATAACAAGAAATTGGCTATTGCTCCTTTCTTCAT     185

inv3_3'end_PacBio 201 ATTTTTTTATTTAGTACTTTTTTTTTAGCCTTGAGTTTAAATAAATTTTT     250
                      ||||||||||||||||||||||||||||||||||||||||||||||||||
inv3_3'end_Sanger 186 ATTTTTTTATTTAGTACTTTTTTTTTAGCCTTGAGTTTAAATAAATTTTT     236

inv3_3'end_PacBio 251 CTTCTTTTATTTCTTTCTATTTTTTAAGAAATAAATAATGGAATTATAAA     300
                      ||||||||||||||||||||||||||||||||||||||||||||||||||
inv3_3'end_Sanger 237 CTTCTTTTATTTCTTTCTATTTTTTAAGAAATAAATAATGGAATTATAAA     287

inv3_3'end_PacBio 301 TACTAGAAATAGAAATTCTGGTAATTTTTAGTGGTAATTTTGACATAGTT     350
                      ||||||||||||||||||||||||||||||||||||||||||||||||||
inv3_3'end_Sanger 288 TACTAGAAATAGAAATTCTGGTAATTTTTAGTGGTAATTTTGACATAGTT     338
```

```
inv3_3'end_PacBio 351 TTAAAATAGAGTTTTGGGGCGGATGTAGCCAAGTGGATAAAGGCGGTGGA    400
                      ||||||||||||||||||||||||||||||||||||||||||||||||||
inv3_3'end_Sanger 339 TTAAAATAGAGTTTTGGGGCGGATGTAGCCAAGTGGATAAAGGCGGTGGA    389

inv3_3'end_PacBio 401 TTGTGAATCCACCACGCGCGGGTTCAATTCCCGTCATTCGCCCATAACCA    450
                      ||||||||||||||||||||||||||||||||||||||||||||||||||
inv3_3'end_Sanger 390 TTGTGAATCCACCACGCGCGGGTTCAATTCCCGTCATTCGCCCATAACCA    440

inv3_3'end_PacBio 451 TAAATAAGATAGAATATGGGATTTTCTAAGATTCTACTATGTGAAATTCG    500
                      ||||||||||||||||||||||||||||||||||||||||||||||||||
inv3_3'end_Sanger 441 TAAATAAGATAGAATATGGGATTTTCTAAGATTCTACTATGTGAAATTCG    491

inv3_3'end_PacBio 501 AATGGGTTGCCCGGGACTCGAACCCGGAACTAGTCGGATGGAGTAGAGAA    550
                      ||||||||||||||||||||||||||||||||||||||||||||||||||
inv3_3'end_Sanger 492 AATGGGTTGCCCGGGACTCGAACCCGGAACTAGTCGGATGGAGTAGAGAA    542

inv3_3'end_PacBio 551 TTTCCTTGTAAAAAAAAAAGGCAAAAAATCCCTCCCCAAACCGTGCTTGC    600
                      ||||||||||||||||||||||||||||||||||||||||||||||||||
inv3_3'end_Sanger 543 TTTCCTTGTAAAAAAAAAAGGCAAAAAATCCCTCCCCAAACCGTGCTTGC    593

inv3_3'end_PacBio 601 ATTTTTCATTGCACACGGCTTTCCCTATGTATACATCTAAAAGCCCGTTC    650
                      ||||||||||||||||||||||||||||||||||||||||||||||||||
inv3_3'end_Sanger 594 ATTTTTCATTGCACACGGCTTTCCCTATGTATACATCTAAAAGCCCGTTC    644

inv3_3'end_PacBio 651 GTTCCCTACTTAGACCTAGGCGGAACTCCTACTCCTAAGAAAGGTGAATA    700
                      ||||||||||||||||||||||||||||||||||||||||||||||||||
inv3_3'end_Sanger 645 GTTCCCTACTTAGACCTAGGCGGAACTCCTACTCCTAAGAAAGGTGAATA    695

inv3_3'end_PacBio 701 CTTAGTTGCTCAATCATTACTCGTATATTGGATGAACATTTCATAATCCA    750
                      ||||||||||||||||||||||||||||||||||||||||||||||||||
inv3_3'end_Sanger 696 CTTAGTTGCTCAATCATTACTCGTATATTGGATGAACATTTCATAATCCA    746

inv3_3'end_PacBio 751 AATGAAGAAATTTCGTTTCTTTTTTATTTGTTATCCTATAAATAGAAATA    800
                      ||||||||||||||||||||||||||||||||||||||||||||||||||
inv3_3'end_Sanger 747 AATGAAGAAATTTCGTTTCTTTTTTATTTGTTATCCTATAAATAGAAATA    797

inv3_3'end_PacBio 801 ATAGAGAAAGAATCTCCATTTAAAAGTTTTCATCACCAATTCTTCCTATC    850
                      ||||||||||||||||||||||||||||||||||||||||||||||||||
inv3_3'end_Sanger 798 ATAGAGAAAGAATCTCCATTTAAAAGTTTTCATCACCAATTCTTCCTATC    848

inv3_3'end_PacBio 851 CTAATTGGGCATATC    865

inv3_3'end_Sanger     ---------------
```

1g. Alignment of the PacBio sequence (in red) with the respective amplicon sequence obtained by the Sanger method (in dark blue). The end of inversion 3 (in bold) and the flanking region are shown. Nucleotides highlighted in yellow indicate primer annealing sites.
